# Differential ACPA Binding to Nuclear Antigens Reveals a PAD-Independent Pathway and a Distinct Subset of Acetylation Cross-Reactive Autoantibodies in Rheumatoid Arthritis

**DOI:** 10.3389/fimmu.2018.03033

**Published:** 2019-01-04

**Authors:** Katy A. Lloyd, Gustaf Wigerblad, Peter Sahlström, Manasa G. Garimella, Karine Chemin, Johanna Steen, Philip J. Titcombe, Bianka Marklein, Diana Zhou, Ragnhild Stålesen, Elena Ossipova, Christina Lundqvist, Olov Ekwall, Johan Rönnelid, Daniel L. Mueller, Mikael C. I. Karlsson, Mariana J. Kaplan, Karl Skriner, Lars Klareskog, Fredrik Wermeling, Vivianne Malmström, Caroline Grönwall

**Affiliations:** ^1^Center for Molecular Medicine, Division of Rheumatology, Department of Medicine, Karolinska Institutet, Karolinska University Hospital, Stockholm, Sweden; ^2^Systemic Autoimmunity Branch, Intramural Research Program, National Institute of Arthritis and Musculoskeletal and Skin Diseases, National Institutes of Health, Bethesda, MD, United States; ^3^Department of Medicine, Charité University Hospital, Berlin, Germany; ^4^Department of Microbiology, Tumor and Cell biology, Karolinska Institutet, Stockholm, Sweden; ^5^The Center for Immunology, University of Minnesota Medical School, Minneapolis, MN, United States; ^6^Department of Rheumatology and Inflammation Research, Institute of Medicine, Sahlgrenska Academy, University of Gothenburg, Gothenburg, Sweden; ^7^Department of Pediatrics, Institute of Clinical Sciences, Sahlgrenska Academy, University of Gothenburg, Gothenburg, Sweden; ^8^Department of Immunology, Genetics and Pathology, Uppsala University, Uppsala, Sweden

**Keywords:** anti-citrullinated protein autoantibodies, acetylation, rheumatoid arthritis, anti-CCP, PAD4, apoptosis, neutrophil extracellular traps (NETs), ANA

## Abstract

Rheumatoid arthritis (RA) associated anti-citrullinated protein autoantibodies (ACPA) target a wide range of modified proteins. Citrullination occurs during physiological processes such as apoptosis, yet little is known about the interaction of ACPA with nuclear antigens or apoptotic cells. Since uncleared apoptotic cells and neutrophil extracellular trap (NET) products have been postulated to be central sources of autoantigen and immunostimulation in autoimmune disease, we sought to characterize the anti-nuclear and anti-neutrophil reactivities of ACPA. Serology showed that a subset of anti-CCP2 seropositive RA patients had high reactivity to full-length citrullinated histones. In contrast, seronegative RA patients displayed elevated IgG reactivity to native histone compared to controls, but no citrulline-specific reactivity. Screening of 10 single B-cell derived monoclonal ACPA from RA patients revealed that four ACPA exhibited strong binding to apoptotic cells and three of these had anti-nuclear (ANA) autoantibody reactivity. Modified histones were confirmed to be the primary targets of this anti-nuclear ACPA subset following immunoprecipitation from apoptotic cell lysates. Monoclonal ACPA were also screened for reactivities against stimulated murine and human neutrophils, and all the nuclear-reactive monoclonal ACPA bound to NETs. Intriguingly, one ACPA mAb displayed a contrasting cytoplasmic perinuclear neutrophil binding and may represent a different NET-reactive ACPA subset. Notably, studies of CRISPR-Cas9 PAD4 KO cells and cells from PAD KO mice showed that the cytoplasmic NET-binding was fully dependent on PAD4, whilst nuclear- and histone-mediated NET reactivity was largely PAD-independent. Our further analysis revealed that the nuclear binding could be explained by consensus-motif driven ACPA cross-reactivity to acetylated histones. Specific acetylated histone peptides targeted by the monoclonal antibodies were identified and the anti-modified protein autoantibody (AMPA) profile of the ACPA was found to correlate with the functional activity of the antibodies. In conclusion, when investigating monoclonal ACPA, we could group ACPA into distinct subsets based on their nuclear binding-patterns and acetylation-mediated binding to apoptotic cells, neutrophils, and NETs. Differential anti-modified protein reactivities of RA-autoantibody subsets could have an important functional impact and provide insights in RA pathogenesis.

## Introduction

In rheumatoid arthritis (RA), the production of anti-citrullinated protein autoantibodies (ACPA) is a distinct disease feature which is used for classification of seropositive RA, and where presence of ACPA associates with increased disease severity and worse prognosis [reviewed in 1]. Recent studies suggest that ACPA may directly play an active role in RA pathogenesis, as ACPA have been shown to mediate bone loss, pain, and enhance arthritis *in vivo* (2–6), as well as inducing pro-inflammatory events in different *in vitro* cell systems (3, 4, 7–11). Citrullination involves the post-translational modification of arginine residues to citrulline by a family of enzymes referred to as peptidylarginine deiminases (PAD), which are involved in several physiological processes including gene regulation, cell differentiation, and apoptosis (12). Of particular interest for RA, citrullination associated with PAD2 and PAD4 expression is present in different inflammatory processes, and is also found in the inflamed RA synovium (13, 14). PAD-mediated citrullination of nuclear antigens such as histones has previously been reported to play an essential role in the unique form of cell death known as neutrophil extracellular trap formation (NETosis) (15, 16), and it has been postulated that enhanced NET production could provide an important source of autoantigens within the inflamed joints of RA patients (7).

In the clinic, the presence of ACPA IgG in the serum of RA patients can be captured using synthetic cyclic citrullinated peptide (CCP2/CCP3) assays. However, serum ACPA IgG can react with peptides derived from many different citrullinated proteins including α-enolase, filaggrin, vimentin, fibrinogen, and histones (17–21). When evaluating the fine-specificity of monoclonal ACPA derived from memory B cells and plasma cells from RA patients it was recently shown that individual ACPA mAbs display remarkable cross-reactivity to different citrullinated peptides and proteins (5, 10, 11, 22, 23). Hence, ACPA mAbs bind to consensus citrulline motifs in peptides rather than specific proteins, albeit with different clones exhibiting distinct peptide reactivity profiles (5, 10). Despite these studies, it is still unclear which citrullinated targets may mediate the pathogenic effects of these cross-reactive ACPA and to which extent monoclonal ACPA displaying different fine-specificity profiles are able to mediate distinct functional effects.

The majority of monoclonal ACPA investigated to date are reported to be encoded by highly somatic hypermutated Ig variable genes (5, 10, 11, 24, 25) and display hypermutation driven variable region glycosylation (25–27), which together are two features that represent the most prominent ACPA characteristics. Since ACPA are present before clinical arthritis and synovitis (28–30), it seems plausible that the process of somatic mutation and selection of certain ACPA-positive B cells progresses over during a long time before onset of arthritis. It is therefore imperative to understand more of which targets and specific BCR features that are most critical in the selection of the autoreactive B cells, in the early phase of autoimmunity, as well as in the pathogenic escalation to chronic disease.

Nuclear antigens generated during cell death have previously been implicated in autoimmune and inflammatory diseases. These autoantigens are postulated to be exposed either due to impaired efferocytosis of apoptotic cells or increased activation of neutrophils with resulting NETosis. Herein, we investigate the interaction between monoclonal ACPA and nuclear antigens, in order to contribute to the understanding of the triggering mechanisms of the autoreactivity and pathogenic roles of different ACPA. Our results highlight a novel interaction between ACPA and apoptotic cells that overlaps with NET-binding. We identify a distinct subset of ACPA with an anti-modified protein autoantibody (AMPA) profile which drive these interactions and demonstrate that the recognition of the nuclear targets of these cross-reactive ACPA were selectively due to binding to specific acetylated histone epitopes.

## Methods

### Clinical Samples

Serum samples were obtained from 243 RA patients and 157 population-based controls from the Epidemiological Investigation of Rheumatoid Arthritis (EIRA) study (31). All patients fulfilled the 1987 ACR RA classification criteria (32) and were subgrouped as ACPA seropositive (193 subjects) or seronegative (50 subjects) using the anti-CCP2 assay (CCPlus, Euro Diagnostica). Patient samples have previously been screened for anti-citrullinated peptide fine-specificities with an antigen microarray multiplex assay, based on Phadia's ImmunoCAP ISAC system, as previously described (33). Peripheral blood mononuclear cells (PBMC) for *in vitro* assays were isolated from heparinized blood from healthy volunteers. The local ethics committee at Karolinska University Hospital approved the study, and all the experiments were performed according to good clinical practice and good laboratory practice. All study subjects gave informed consent to participate.

### Detection of Antibodies to Citrullinated and Native Histone 2B in RA Serum

Serum levels of autoantibodies to citrullinated and native full-length histone were measured by ELISA in patients and controls. Since histone 2B amino acid sequence is conserved between species, purified bovine protein was used in the assay. Briefly, 2 mg/ml histone 2B (His2B, Immunovision) was citrullinated in solution for 2 h at 37°C in 100 mM Tris, 10 mM CaCl_2_, 5 mM DTT, PAD4 0.75 U/mg histone protein (Calbiochem). Native His2B was prepared using the same protocol but without the PAD enzyme. Antigens were then buffer exchanged to PBS using slide-a-lyzer dialysis cassettes (Life Technologies). Citrullinated or native His2B were coated at 3 μg/ml in 100 mM carbonate buffer (pH 9.6) into separate wells on the same ELISA plate (using Corning high binding ELISA plates), and subsequently blocked with 3% BSA (Sigma-Aldrich) in PBS. Serum samples were evaluated at 1:50 dilution in sample buffer (2% BSA, 5% normal donkey sera (Jackson Immunoresearch), 0.1% Tween20, and 0.3 M NaCl in PBS). Reactivity was detected with goat F(ab')_2_ anti-human IgG(Fc)-HRP in PBS (Jackson Immunoresearch), developed with TMB substrate (Biolegend), and stopped with 2*N* H_2_SO_4_. An RA reference sample was used as a positive control on the cit-His2B surface to generate a standard curve and extrapolate reactivity values in relative units (RU)/ml. Cutoff for positivity was set based on 95th percentile on the population controls for anti-native His2B, anti-citrullinated His2B, or normalized reactivity [IgG anti-cit-His2B (RU/ml) divided by IgG anti-nat-His2B (RU/ml)].

### ACPA Monoclonal Antibodies

ACPA and control mAbs were derived from single B cells from different B cell populations isolated from RA patients, as previously reported (5, 10, 26, 34–36). Four monoclonal ACPA were derived from synovial plasma cells (1325:01B09, 1325:04C03, 1325:05C06, 1325:07E07) (10). Six ACPA were derived from antigen-tetramer sorted circulating memory B cells, using cyclic citrullinated alpha enolase peptide (CEP) tetramers (clones 37CEPT1G09, 37CEPT2C04), or cyclic citrullinated filaggrin peptide 1 (CFC1) tetramers (clones 14CFCT2D09, 14CFCT2H12, 14CFCT3G09, 62CFCT1E04) (5). RA synovial memory B cell-derived mAbs with no identified specific binding, were used as negative controls (1362:01E02, 1276:01G09). Immunoglobulin genes were cloned using established methods (35, 37) and expressed as human IgG1 or chimeric murine IgG2a in Expi293 cells (Life Technologies). Additionally, 1325:01B09 was mutated to generate a heavy chain with null-binding to Fcγ receptors following a previously reported strategy with validated G236R and L328R amino acid replacements (GRLR hIgG1, template plasmid was a kind gift from Dr. Stylianos Bournazos and Dr. Jeffrey V. Ravetch, Rockefeller University) (38). Quality control of the expressed IgG included SDS-PAGE, size exclusion chromatography aggregation tests, endotoxin test, and citrullinated antigen-specificity ELISA. hIgG1 mAbs were conjugated with DyLight 650 (Thermo Fisher Scientific) for flow cytometry analysis of citrullination in murine neutrophils, and biotinylated with EZ-Link Sulfo-NHS-LC-Biotin (Thermo Fisher) for human thymus immunofluorescence, according to manufacturer's instructions.

### Screening of Monoclonal ACPA Binding to Citrullinated Full-Length Proteins

Human recombinant histones (1, 3, and 4), hnRNPs (A3, B1, D, DL), and vimentin were expressed and purified as previously described (39). Antigens solubilized in 8M urea were coated on Nunc Maxisorb 96-well plates. Plates were blocked with 5% milk powder in PBS, and washed with PBS/0.1% Tween. Plate-bound antigens were citrullinated using 150 mU/ml PAD2 or PAD4 (Modiquest) in buffer (50 mM Tris, 10 mM CaCl_2_, 1 mM DTT). Binding of monoclonal ACPA (1 μg/ml) to different citrullinated antigens were determined with anti-hIgG Fc-HRP (Sigma-Aldrich) and developed with TMB (Seramun Diagnostica).

### Monoclonal ACPA Binding to Modified Peptides

ACPA mAb reactivity to the citrulline (cit) peptides cit-Vim60-75 (biotin-HQCVYAT-Cit-SSAV-Cit-L-Cit-SSVPC), Cit-Fibα563-583 (biotin-HQCHHPGIAEFPS-Cit-GKSSSYSKQFC), or acetyl-lysine (Ac) peptides Ac-His2B:6-22 (biotin-HQCSAPAPK-Ac-GSKKAVTKAQC), Ac-His4:1-23 (biotin-SGRG-Ac-GG-Ac-GLG-Ac-GGA-Ac-RHRKVLR) or native arginine (Nat-Vim60-75 and Nat-Fibα563-583) or lysine (Nat-His2B:6-22 and Nat-His4:1-23) counterparts was detected by ELISA. Briefly, high-binding half-area ELISA plates were coated with neutravidin 3 μg/ml (Life Technologies), blocked with 3% BSA, and biotinylated peptides were captured at 1 μg/ml followed by incubation with human mAbs at 5 μg/ml in sample buffer (2% BSA, 5% normal donkey sera (Jackson Immunoresearch), 0.1% Tween20, and 0.3 M NaCl in PBS). Reactivity was detected with goat F(ab')_2_ anti-human IgG(Fc)-HRP in PBS (Jackson Immunoresearch) and TMB substrate (Biolegend).

### Detecting ACPA Binding to Apoptotic Cells

Murine thymocytes were isolated from thymus tissue, strained through a 70 μM cell strainer, and re-suspended to 2 × 10^6^ cells/ml in RPMI 1640 with 10% ultra-low IgG FBS (Life Technologies), 1% penicillin/streptomycin and 1% L-glutamine (Life Technologies). Apoptosis was induced by incubation with 10 μM dexamethasone (Sigma-Aldrich) at 37°C for 4 h, as previously described (40). In Jurkat (human T-cell line) cells, apoptosis was induced with 100 ng/ml anti-CD95/Fas (EOS9.1, Biolegend) for 2 h or 25 μM etoposide (Sigma-Aldrich) overnight at 37°C. Thereafter, cells were incubated with mAbs (10 μg/ml) in 3% BSA in PBS for 1 h on ice, and detected with biotinylated goat anti-human IgG (Jackson Immunoresearch) and streptavidin-PE (BD Biosciences). Apoptosis was confirmed by staining cells with Annexin V and 7AAD (BD Biosciences) according to the manufacturer's instructions. Data were acquired using a BD FACSVerse™ flow cytometer and analyzed with FlowJo software (Tristar). For PAD or HDAC inhibition, Jurkats were pre-incubated with either 20 μM Cl-amidine (Cl-A, Calbiochem) or 100 ng/ml trichostatin A (TSA, Sigma-Aldrich), respectively, for 3 h at 37°C before stimulation of apoptosis with 25 μM etoposide (Sigma-Aldrich) overnight. Whole cell lysates were prepared in RIPA buffer (Sigma-Aldrich) with Complete protease inhibitor cocktail (Roche) and then 20 μg protein per lane was separated on SDS-PAGE under reducing conditions using NuPAGE bis-tris gels (4–12%) and MES-SDS running buffer (Life Technologies) followed by transfer to PVDF membranes according to the manufacturer's instructions (Life Technologies). ACPA binding was detected in the Western blot with 5 μg/ml hIgG1 followed by rabbit anti-human IgG(Fc)-HRP in PBS (Jackson Immunoresearch) and chemiluminescence developing with Clarity Western ECL substrate (Bio-Rad). Acetylation of histone 2B was detected with rabbit anti-AcH2B-K12 (Cell Signaling) and anti-rabbit-HRP (Cell Signaling). Citrullination in cell lysates was detected using a rhodamine based citrulline-specific probe (Cayman Chemicals). The probe was solubilized in acetonitrile:water (2:1) and 3 μg probe were added to 40 μL cell lysates pre-treated with 20% trichloroacetic acid (at 1 mg/ml total protein). Samples were incubated 30 min followed by centrifugation at 15,000 g for 5 min. Pellets were washed twice in ice-cold acetone and resuspended in LDS loading dye with reducing agent and SDS-PAGE separated. In-gel fluorescence in the gels was visualized with the ChemiDoc MP system (Bio-Rad). To validate protein loading, the gels were subsequently stained with Coomassie (SimplyBlue SafeStain, Life Technologies).

### Immunoprecipitation of Apoptotic Cell Antigens

Immunoprecipitations were used to detect ACPA-binding proteins in apoptotic cells. Apoptosis was induced in Jurkat cells with 25 μM etoposide (Sigma-Aldrich) for 16 h at 37°C. Cells were washed with PBS and resuspended (1 × 10^7^/ml) in TNI lysis buffer (50 mM Tris, 250 mM NaCl, 0.5% Igepal CA-630) supplemented with Complete protease inhibitor cocktail (Roche), and incubated for 30 min on ice. After centrifugation, the lysate soluble phases were pre-cleared by 1 h incubation with protein G resin (GE Healthcare), followed by addition of 3 μg ACPA or control hIgG1 to 400 μl lysate, and incubation at 4°C overnight. Antibody-antigen complexes were captured with 40 μl protein G resin, washed twice with TNI including detergent and without detergent, and either eluted with four times 50 μl 0.5 M ammonium hydroxide and dehydration by SpeedVac or separated on SDS-PAGE under reducing conditions using NuPAGE bis-tris gels and MES-SDS running buffer (Life Technologies). SimplyBlue SafeStain (Life Technologies) stained bands were excised and subjected to mass spectrometry analysis.

### In-gel Digestion and LC-MS/MS Analysis

Excised gel bands were destained in 100 mM ammonium bicarbonate at 37°C for 30 min. In-gel digestion was performed as previously described (41). Peptides were extracted from the gel bands using subsequently 20% acetonitrile (ACN) in 0.1% formic acid (FA), 50% ACN in 0.1% FA, and 100% ACN. Collected supernatants were dried using SpeedVac and reconstituted in 3% ACN in 0.1% FA.

Peptide identification was performed using Dionex Ultimate 3,000 nano-LC system coupled via electrospray ion source (Thermo Fisher Scientific) to a Q-Exactive Orbitrap (Thermo Fisher Scientific). Peptides were trapped onto Acclaim™ PepMap™ 100 C18, 3 μm, 100 Å trap column, 75 μm i.d. × 2 cm, and separated on nanoEase M/Z™ HSS T3, 100 Å, 1.8 μm, 75 μm × 250 mm column (Waters). Peptides were eluted with a linear gradient of 5–45% of buffer B (95% v/v acetonitrile, 0.1% v/v FA) at flow rate 250 nl/min at RT. Data acquisition was performed with a Top10 data-dependent MS/MS scan. Scan range was set to 300–1,600 m/z, with a maximum injection time of 250 ms and resolution of 70,000 at m/z 400. Fragmentation of precursor ions was performed at 28 eV of collision energy. MS/MS scans were performed at resolution of 17,500 with an ion target value of 2 × 10^5^ and the injection time of 200 ms. MS raw files were analyzed by MaxQuant software (Version 1.5.5.1) (42) and searched against human Uniprot/Swissprot database (release-2018_04), with enzyme specificity set to “Trypsin” allowing two missed cleaved sites. Carbamidomethylation was set as fixed modifications and deamidation of R, Q, and N set as variable modifications. Precursor mass deviation was set to 10 ppm and fragment mass deviation to 20 ppm. The false discovery rate was set to 0.01.

### Solid-Phase Immune Complex Assay

Monoclonal ACPA were used to generate plate-bound Cit-His2B immune complexes, as previously described with minor modifications (43). Briefly, 10 μg/ml PAD4-citrullinated His2B or control native histone proteins were coated to MaxiSorp ELISA plates (Nunc) overnight at 4°C. Plates were blocked with 1% ultra-low IgG FBS in PBS, then incubated with monoclonal antibodies (10 μg/ml) for 1 h at 37°C. PBMC were isolated from healthy donors with Ficoll-Paque Plus (GE Healthcare) separation and added to the wells (10^6^ cells/ ml) in RPMI 1640 medium with 5% ultra-low IgG FBS, 1% penicillin/streptomycin, 1% HEPES, and 12.5 μg/ml polymyxin B sulfate (Sigma-Aldrich). TNF-α or IL-8 release in supernatants after 20 h was measured by ELISA (Biolegend), using recombinant IL-8 or TNF-α as standards (Biolegend).

### Investigation of ANA Reactivity

Monoclonal ACPA (5 μg/ml) and patient sera (1:40 dilution) were screened for anti-nuclear autoantibody reactivities (ANA) using the NOVA® Lite HEp-2 ANA substrate slide kit, according to the manufacturer's guidelines (Inova Diagnostics). Staining patterns were designated based on the guidelines provided by the International Consensus on Antinuclear Antibody Pattern (ICAP) available at the website www.anapatterns.org (44). ACPA mAbs were also assayed at 5 μg/ml with ANA line blot (EUROLINE ANA profile 3, Euroimmune).

### ACPA Binding to Human Thymus

Thymus tissue was obtained from children undergoing cardiac surgery. Parents gave informed written consent. The study was approved by the Regional Ethical Board at the University of Gothenburg. The tissue was embedded in Optimal cutting temperature compound (OCT) (Histolab Products AB) in isopentane pre-cooled with liquid nitrogen, and 7 μm sections were cut and fixed in cold acetone. After rehydration in PBS and blocking with Protein Block (Dako), tissue sections were stained with biotinylated monoclonal antibodies (5 μg/ml) and anti-cytokeratin 5 (BioLegend), and detected with anti-rabbit-AlexaFluor488, streptavidin-AlexaFluor555, and Hoechst, and mounted with ProLong Gold Antifade mountant (all Life Technologies). For flow cytometry, thymocyte single cell suspensions were prepared by enzymatic (DNase I and Liberase TH, Roche) and mechanical (gentleMACS^TM^ Dissociator, Miltenyi Biotec) treatment. Cells were blocked with Beriglobin (CSL Behring) and stained with biotinylated monoclonal antibodies (10 μg/ml) and Fixable Viability Dye 506 (Life Technologies), followed by Streptavidin APC (BD Bioscience). For intracellular staining, cells were fixed with the Transcription Factor Staining Buffer Set (Life Technologies).

### ACPA Binding to Human Neutrophils and NETs

Enriched human neutrophil fractions were isolated from heparinized blood from two healthy donors, as previously described (45). Briefly, after Ficoll-Paque separation (GE Healthcare), granulocytes were twice treated with red blood cell lysis using ACK lysing buffer (Thermo Fisher Scientific). Primary human neutrophils (0.2 × 10^6^) were cultured in Hanks' balanced salt solution without calcium/magnesium for 4 h on 6 mm coverslips coated with 0.02% poly-L-Lysine (Sigma-Aldrich). Neutrophils were left untreated (medium) or stimulated with ionomycin (1 μM, Calbiochem) or phorbol 12-myristate 13-acetate (PMA) (50 nM, Calbiochem), then fixed with 3.7% formaldehyde and incubated in PBS-glycine (10 mM). Cells were permeabilized (eBioscience, Foxp3 permeabilization buffer), stained with murine chimeric ACPA IgG2a or control mAbs (10 μg/ml), and subsequently with anti-mouse-AlexaFluor488 (Invitrogen) and Hoechst (Thermo Fisher Scientific). Coverslips were mounted onto glass slides using Fluoromount-G (Southern Biotech). Images were acquired using a Leica TCS SP5 and a 63x oil objective. A z-dimension series was taken every 0.2 μm. Images were analyzed with the Fiji software.

### ACPA Binding to Murine Neutrophils and NETs

ECoM-G cells (kind gift from Dr. Mark Kamps, University of California San Diego) (46) were grown in DMEM with 10% FBS (Life Technologies), 4% GM-CSF conditioned medium, 1% penicillin/streptomycin/L-glutamine (Life Technologies), and 1 μM β-estradiol (Sigma Aldrich). To differentiate cells into neutrophils, the β-estradiol was removed and cells were cultured for 5 days until further analysis. Cells were stimulated with ionomycin for 4 h (1 μM, Thermo Fisher Scientific) or a DMSO vehicle for NETosis induction. Ionomycin produced enhanced NET formation compared to PMA in this cell system. Pharmacological inhibition of PAD enzymes was performed by addition of 100 μM Cl-amidine (Cl-A, Calbiochem) during the 5 day differentiation culture. For intracellular staining, cells were fixed and permeabilized using Foxp3 buffer set according to manufacturer's instructions (Thermo Fisher Scientific), stained with 10 μg/ml with DyLight-650-conjugated ACPA hIgG1 or control monoclonal antibody, and co-stained with anti-Ly6/G. Flow cytometry data was acquired using a BD Accuri instrument (BD Biosciences). For immunohistochemistry, ECoM-G cells were grown on coverslips (Falcon, Corning), fixed using 4% PFA, permeabilized (0.2% TritonX), and stained with ACPA (10 μg/ml), followed by detection with Alexa-488 goat anti-human IgG (Life technologies), and DAPI. Images were acquired as above. Guide RNA (gRNA) targeting the *Padi4* gene (CTGGACAAGTCTAACCCGGT) was selected using the Green listed software (47) and the Brie library (48), and were cloned into the vector px459 (kind gift from Dr. Feng Zhang, Addgene plasmid #62988) (49). ECoM-G cells were electroporated (Neon System, Life Technologies) with the plasmid and rested for 24 h prior to puromycin selection (10 μg/ml) for 48 h. Limiting dilution were then used to generate single cells in 96-well plates, which were sequenced to determine successful mutation of the PADI4 gene. Loss of protein was validated using separation of cell extracts on SDS-PAGE (NuPage 4–12% Bis Tris, Life Technologies), transfer to PVDF membrane (Bio-Rad Trans Blot Turbo), and detection with an anti-PAD4 specific antibody (ab214810, Abcam) with anti-β-actin as loading control. For staining of primary bone marrow cells, tibial bone marrow was isolated from 8–12-week-old female Balb/c, FVB, Pad2^−/−^, or Pad4^−/−^ mice, strained through a 70 μM cell strainer, and stained with biotinylated lineage panel (Miltenyi Biotech) or CD11b (Cell Signaling) and Ly6G (Cell Signaling), in addition to 10 μg/ml DyLight-650-conjugated hIgG1 ACPA. Bone marrow from WT FVB, Pad2^−/−^, or Pad4^−/−^ mice was purified using magnetic beads (neutrophil isolation kit, Miltenyi Biotec) according to manufacturer's instructions. One million purified neutrophils were resuspended in DMEM with 1 μM ionomycin or vehicle for 1 h, then processed for analysis. Detection of citrullinated proteins in cell lysates using a rhodamine based citrulline-specific probe (Cayman Chemicals) was performed as described above.

### *In vivo* Administration of Apoptotic Cells

For generating apoptotic cells, thymocytes from 4- to 5-wk-old sex-matched C57BL/6 mice were cultured for 6 h in complete RPMI 1640 media (Life Technologies) plus 1 μM dexamethasone (Sigma-Aldrich) at 37°C, as described previously (50). Mice were injected i.v. with 10 × 10^6^ apoptotic cells weekly for 4 weeks. The mice received murine chimeric IgG2a ACPA, 1 mg per mouse, together with apoptotic cells in the first and third injections. Apoptotic cells were pre-incubated with ACPA mAbs (5 mg/ml) for 30 min before the injections. Four group of mice were investigated, receiving: PBS and no apoptotic cells; only apoptotic cells, apoptotic cells in combination with the ACPA 1325:01B09 and apoptotic cells in combination with the ACPA 1325:04C03. Mice were bled every week and serum was collected. Antibodies against DNA were measured as described previously (50). Briefly, ELISA plates were coated with methylated BSA plus calf thymus DNA (Sigma-Aldrich). Antigen-reactive IgG in the serum was measured with alkaline phosphate-conjugated anti-mouse IgG antibody (Southern Biotech). Antibodies against Ro-52, Ro-60, SmD1, and SS-B were measured using commercial kits (Signosis, Inc), following manufacturer's instructions. Reactivity to citrullinated full-length proteins were assessed by ELISA, as injected IgG2a antibodies could be discriminated from endogenously produced IgG2 owing to the C57BL/6 IgG2c restriction. Briefly, rabbit PAD citrullinated recombinant human vimentin (produced in-house), PAD4 citrullinated purified human fibrinogen (Sigma Aldrich), or PAD4 citrullinated histone 2B were coated at 3 μg/ml, wells were blocked with 3% BSA and reactivity was assessed in serum at 1:100 dilution. HRP conjugated subclass specific detection antibodies (Southern Biotech) were used to measure either the injected antibodies (anti-IgG2a) or the induced response (anti-IgG1, anti-IgG2c). Anti-CCP3 reactivity was determined using commercial ELISA plates (Inova Diagnostics) but with the murine subclass specific detection reagents as above.

### Statistical Analysis

Statistical analysis of serological data was performed with Prism (Graphpad Softwares). Serological measurements were found to have a non-Gaussian distribution and unequal variances. Hence, *t*-test with Welch correction for unequal standard deviation was used for comparing continuous variables between groups and Spearman analysis for correlation analysis. Experimental data points from *in vitro* assays were compared with Student's *t*-test. *P* < 0.05 were considered significant.

## Results

### Reactivities to Native and Citrullinated Histones in RA Patients

**Figure 1:**
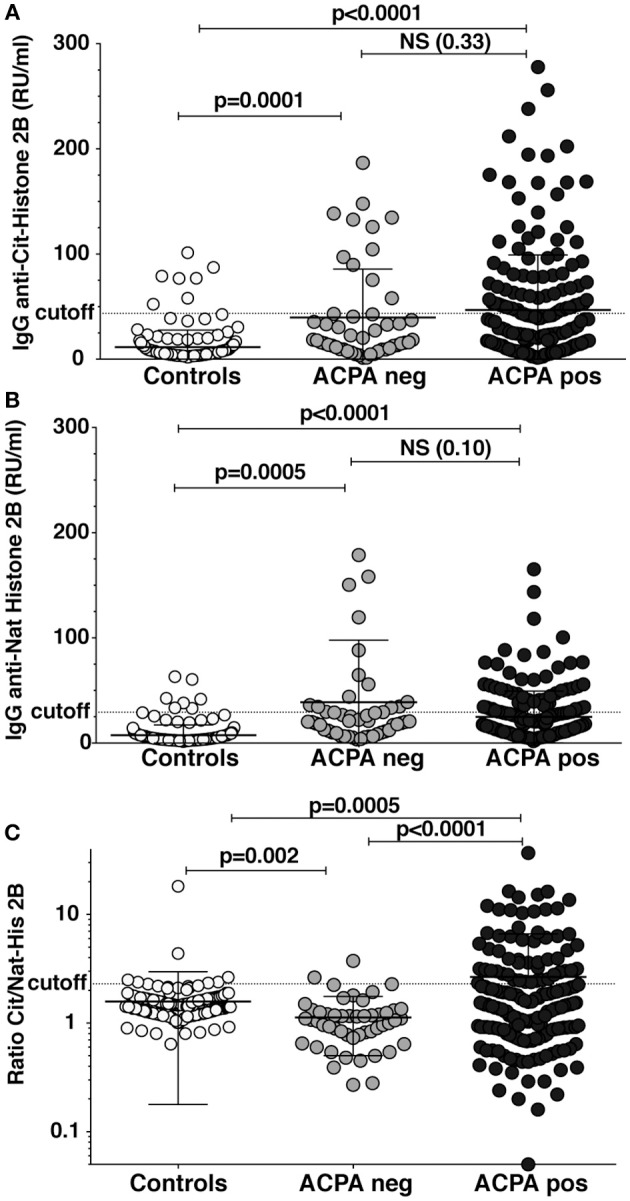
Reactivity against citrullinated full-length histone in RA patients. Serum levels of IgG binding to full-length citrullinated histone **(A)** and native histone **(B)** was determined by ELISA in 193 CCP2 positive RA patients, 50 CCP2 negative, and 157 healthy population controls using purified bovine histone 2B citrullinated in solution with PAD4. **(C)** The IgG anti-citrullinated histone 2B was normalized for native histone reactivity in the patients (Ratio Cit/Nat-His2B). Cutoff for positivity highlighted in the figure was determined by the 95th percentile of the population controls. *P*-values are presented from *t*-test with Welch correction.

Autoantibodies to full-length native and citrullinated histone (His2B) were investigated using sera from ACPA-positive and ACPA-negative RA patients and population controls. As shown in Figure 1, we found increased levels of antibodies to both native and citrullinated His2B protein in both ACPA-negative and ACPA-positive patients as compared to controls. However, only the ACPA-positive patients had citrulline-specific reactivity, which was evident when adjusting the citrulline reactivity against native reactivity (ratio anti-cit/natHis2B). Significantly raised IgG cit-His2B/nat-His2B serum reactivities were seen in the ACPA-positive patients compared to ACPA-negative patients (*p* < 0.0001) or controls (*p* = 0.0005) (Figure 1C). Overall, 29% of ACPA-positive patients were positive for reactivities against citrullinated His2B after normalization for anti-native His2B (Table 1). Any detectable reactivity in the citrullinated histone assay among ACPA negative patients could be explained by reactivity to native histone epitopes, something that could also be visualized when analyzing the correlation between native and citrulline reactivities (Supplementary Figure 1). It is also possible that there are reactivities to cit-His epitopes that are not captured by the CCP2 test, similar to what has been seen for some other citrulline fine-specificities (17). One of the population controls, although without RA diagnosis, was found to have high anti-CCP2 levels that explained the observed anti-cit-His2B reactivity in this individual. The higher levels of reactivity to native histone observed in ACPA-negative patients compared to controls were reflected by a significantly lower anti-cit/nat-His2B ratio (*p* = 0.002, Figure 1C). IgG anti-cit-His2B levels showed a significant negative correlation with age in RA (*p* = 0.03, *r* = −0.14) and positive correlation with IgG anti-CCP2 levels (*p* < 0.0001, *r* = 0.3; Supplementary Table 1). No significant associations with CRP levels or DAS28 disease activity score were observed neither for IgG anti-cit-His2B levels, nor for anti-cit/nat normalized values. When comparing fine specificities, anti-cit-His2B and the anti-cit/nat-His2B ratio correlated with citrullinated vimentin (*p* < 0.001), fibrinogen (*p* < 0.0001), and α-enolase (CEP-1, *p* < 0.0001) peptides (Supplementary Table 1).

**Table 1:** IgG reactivities to full-length citrullinated histone 2B in rheumatoid arthritis patients.

	**Population controls (*n* = 157)**	**ACPA negative RA (*****n*** **=** **50)**		**ACPA positive RA (*****n*** **=** **193)**		**ACPA pos vs. ACPA neg RA**
	**Frequency/mean**	**Frequency/mean**	***p*-value#**	**Frequency/mean**	***p*-value#**	***p*-value#**
Age	55.4 ± 10.5	51.1 ± 13.6	0.04	49.9 ± 11.8	<0.0001	NS (0.58)
Females	71% (111/157)	60% (30/50)	NS (0.16)	75% (145/193)	NS (0.40)	NS (0.051)
DAS28	N/A	5.3 ± 1.1 (27)	N/A	4.9 ± 1.4 (123)	N/A	NS (0.12)
CRP	N/A	22.7 ± 22.3 (31)	N/A	28.1 ± 31.0 (132)	N/A	NS (0.27)
IgG anti-Cit His2B (RU/ml) mean ± SD	11.6 ± 16.1	39.6 ± 46.1	0.0001	46.9 ± 52.2	<0.0001	NS (0.33)
Positive IgG anti-Cit His2B (>43.5 RU/ml) % (n/N)€	4.5% (7/157)	22% (11/50)	0.0005	37% (72/193)	<0.0001	0.046
IgG anti-native His2B (RU/ml) mean ± SD	7.8 ± 9.7	38.9 ± 59.0	0.0005	24.8 ± 24.7	<0.0001	NS (0.10)
Positive IgG anti-native His2B (>29.3 RU/ml) % (n/N)€	4.5% (7/157)	32% (16/50)	<0.0001	28% (55/193)	<0.0001	NS (0.61)
Normalized IgG anti-cit-His2B/nat-His2B mean ± SD	1.58 ± 1.4	1.13 ± 0.62	0.002	2.66 ± 3.96	0.0005	<0.0001
Positive IgG anti cit-His2B/nat-His2B (>2.3) % (n/N) €	4.5% (7/157)	4% (2/50)	NS (1)	29% (55/193)	<0.0001	0.0001

€ *Cutoff was set based on 95th percentile on the population controls*.

# *P-value from 2-sided t-test with Welch's correction for difference in variance*.

Ratio cit/native ACPA positive RA, sensitivity 49%, specificity 89%, OR 7.6 (3.4–17.2)//compared to controls//.

*Ratio cit/native ACPA positive RA, sensitivity 24%, specificity 96% OR 8.5 (2.0–36.2)//compared to ACPA negative//*.

### A Subset of Monoclonal ACPA Have a Binding Profile With High Reactivity to Citrullinated Nuclear Antigens

**Figure 2:**
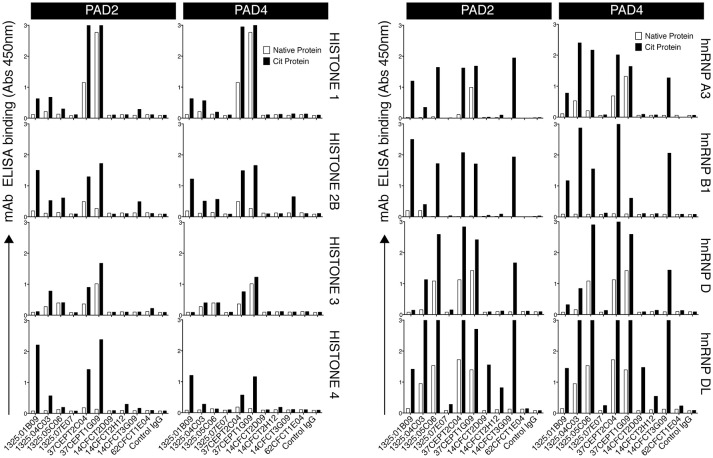
A subset of monoclonal ACPA-IgG exhibit reactivities to citrullinated nuclear antigens. Reactivity of 10 monoclonal ACPA IgG to full-length citrullinated nuclear antigens was evaluated by ELISA using recombinant human antigens (hnRNP A3, hnRNP B1, hnRNP D, hnRNP DL, Histone 1, Histone 3, Histone 4) or purified antigen (Histone 2B) citrullinated on-plate in solid phase under denaturing conditions with either PAD2 or PAD4. Native protein was mock-treated without the enzyme. Human IgG1 was evaluated for binding at 5 μg/ml. The RA-derived mAb 1276:01G09 was used as negative control (control IgG). The figure shows representative data of at least three repeated experiments and bars depicts average value of duplicates.

Among potential nuclear autoantigens, histones, and heterogeneous nuclear ribonucleoproteins (hnRNPs) have been implicated in RA. We therefore investigated the reactivity of 10 RA patient-derived monoclonal ACPA toward full-length citrullinated histones compared to citrullinated hnRNPs. Some ACPA (1325:01B09, 37CEPT2C04, 37CEPT1G09) exhibited broad reactivities for different citrullinated histones and citrullinated hnRNPs (Figure 2), whereas other ACPA bound more to hnRNPs compared to histones (1325:05C06, 14CFCT3G09, 1325:04C03), and one group did not bind any of the investigated antigens. The majority of the monoclonal ACPA were highly specific for citrullinated compared to native proteins and only two clones (37CEPT2C04 and 37CEPT1G09) exhibited a limited cross-reactivity to the native forms of certain full-length antigens (Figure 2). The monoclonal ACPA all displayed anti-CCP2 and anti-CCP3 reactivity at 1,000 ng/ml using commercial assays and the majority also had high reactivity at 100 ng/ml (Supplementary Figure 2), as well as specific reactivity to different citrullinated peptides and no/limited reactivity to arginine peptide versions determined by several antigen microarray platforms as previously published (5, 10, 33). Monoclonal ACPA also bound to the full-length citrullinated cytoplasmic proteins vimentin and α-enolase (Supplementary Figure 3). When comparing the antigens side-by-side, the citrullinated histone ACPA binding strength was equivalent to binding to citrullinated fibrinogen and citrullinated vimentin, and among the different clones the highest cit-histone binding was detected for 1325:01B09 with significant reactivity below 100 ng/ml (Supplementary Figure 4). In general, the clones with high cit-vimentin and cit-α-enolase reactivities showed reduced reactivity to multiple citrullinated histones. No major differences in ACPA reactivities could be detected due to the preference of proteins citrullinated by either PAD2 or PAD4 (Figure 2). In addition, the results were not dependent on which citrullination protocol was used as similar binding was detected with on-plate citrullination compared to in-solution citrullination (Supplementary Figure 4).

### Citrullinated Histone-Containing Immune Complexes Stimulate Innate Immune Responses *in vitro*

**Figure 3:**
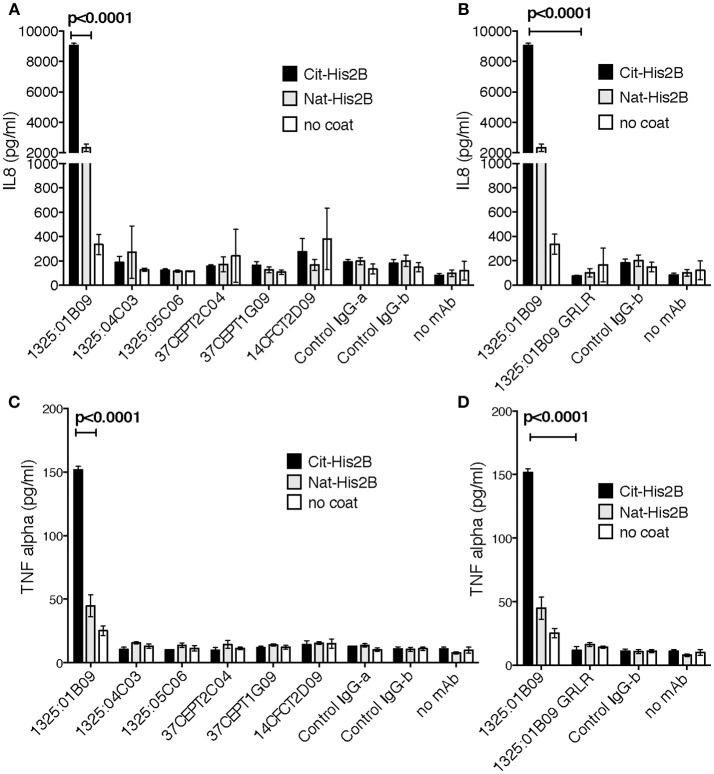
The interaction between citrullinated histone and monoclonal ACPA induce innate responses. The immune stimulatory effect of seven ACPA monoclonal with high cit-histone 2B binding (1325:01B09, 37CEPT1G09, 37CEPT2C04), medium binding (1325:04C03, 1325:05C06, 14CFCT3G09), and no or low binding (14CFCT2D09) was evaluated using plate-bound immune complexes at 10 μg/ml with PAD4 citrullinated histone 2B. Healthy donor PBMC were incubated with the antigen-captured IgG plates for 20 h at 37°C and IL-8 **(A)** and TNF-α **(C)** expression was subsequently assessed in the cell supernatants by ELISA. The importance of FcγR interaction for 1325:01B09-mediated cytokine stimulations was assessed by comparison to the FcγR-null binding genetic variant 1325:01B09 GRLR **(B,D)**. Both recombinant RA derived human IgG (1276:01G09, Control IgG-b) and commercial isotype control IgG (ET901, Biolegend, Control IgG-a) were used as controls. The figure show values in triplicate for one representative donor. Data for multiple donors are available in Supplementary Figure 5. *P*-values are derived from student *t*-test.

Immune complex mediated immune cell activation may play a role in the RA pathogenesis and citrullinated histones have been suggested to be important drivers, hence, we investigated the ability of the monoclonal ACPA in citrullinated His2B immune complexes to induce pro-inflammatory cytokine secretions. For this, freshly isolated but otherwise unstimulated PBMCs were incubated on either native or citrullinated His2B pre-incubated with ACPA hIgG1 mAbs. Interestingly, only one of the citrullinated His2B-binding ACPA (1325:01B09) induced a significant increase in IL-8 and TNF-α (*p* < 0.0001, Figure 3 and Supplementary Figure 5). This activity reflected the 1325:01B09 cit-histone 2B binding level and was dependent on Fcγ-receptor interactions, as a mutated version of 1325:01B09 (GRLR) unable to bind FcγR failed to induce cytokine production (Figure 3). No significant cytokine release was introduced by citrullinated histone antigen alone or by any of the monoclonal ACPA themselves.

### ACPA Bind to Apoptotic Cells and Have ANA Activity

**Figure 4:**
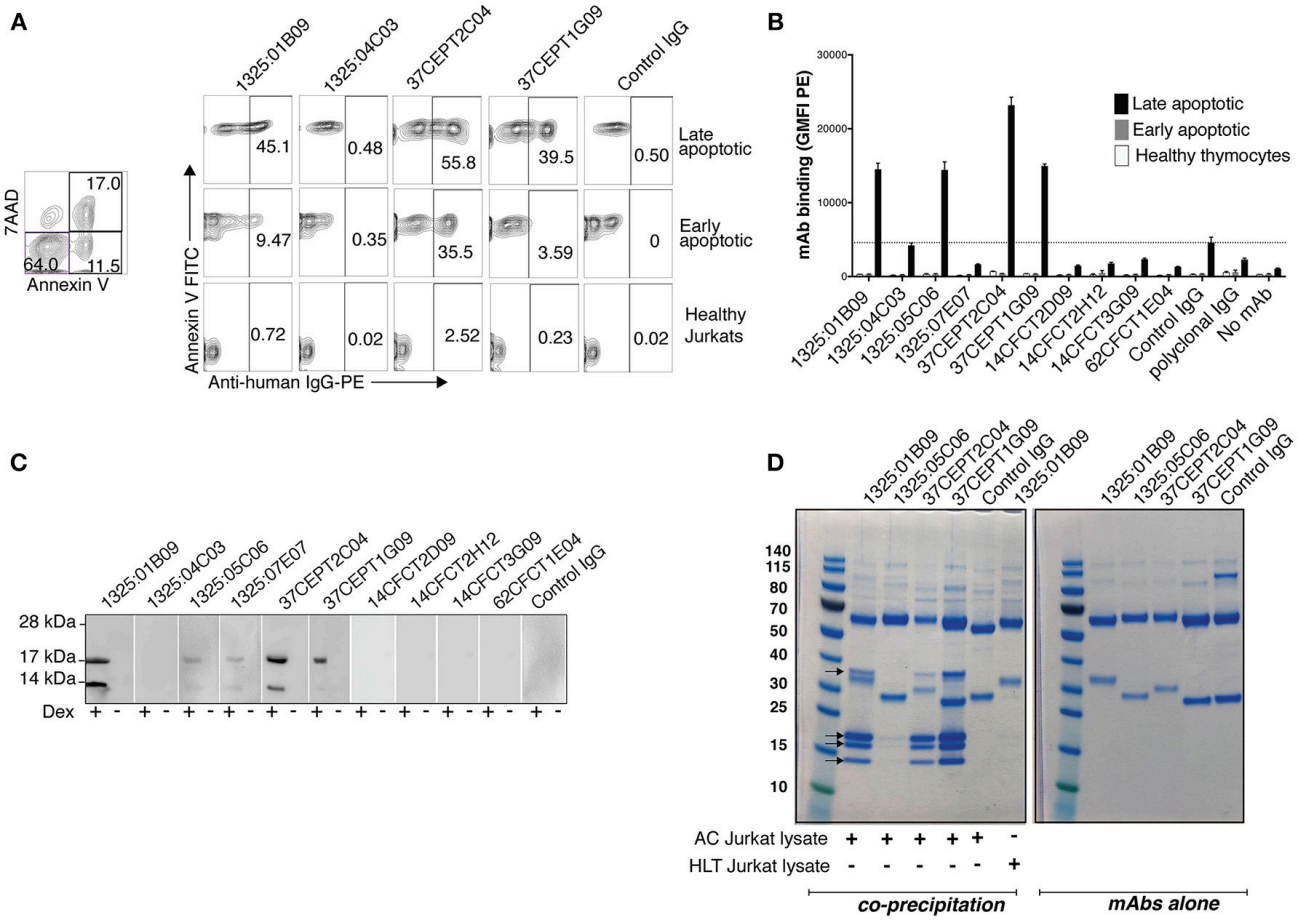
Anti-citrullinated histone ACPA bind apoptotic cells. Flow cytometric analysis revealed that the subset of monoclonal ACPA with citrullinated histone reactivities interacted with apoptotic cells. Binding was assessed by flow cytometry at 10 μg/ml IgG1 using anti-Fas treated human Jurkat T-cells **(A)** or dexamethasone treated primary murine thymocytes **(B)** Binding to late apoptotic cells (7AAD positive, Annexin V positive) was compared to early apoptotic cells (7AAD negative, Annexin V positive) or healthy cells (7AAD negative, Annexin V negative). IgG binding was detected with anti-human IgG PE. A cutoff was set based on the level of binding of the negative control (1362:01E02) to apoptotic cells. **(C)** Immunoblotting of murine thymocyte lysate revealed that these ACPA bound proteins of ~17 kDa and 14 kDa in apoptotic cells (+ dex), which were not observed in the untreated cells (–dex). **(D)** Immunoprecipitation of proteins from apoptotic Jurkat cell lysate (400 μl from 10^7^ cells/ml) with monoclonal ACPA (3 μg) reveal four specific co-precipitated protein bands on SDS-PAGE for 1325:01B09, 37CEPT1G09, 37CEPT2C04, mass spectrometry confirmed that these are different histone proteins. No band was seen for the control non-treated lysates or for a control non-ACPA RA derived mAb (1276:01G09). IgG alone, without cell lysate, are shown in the right panel. Representative figure of three repeat experiments.

Since apoptosis has been implicated in the triggering of autoimmunity and pathogenesis of autoimmune diseases, and is associated with increased citrullination of histones, we investigated whether monoclonal ACPA exhibited reactivities for apoptotic cells. A subset of the monoclonal ACPA (1325:01B09, 37CEPT2C04, 37CEPT1G09, 1325:05C06) was observed to bind both human and murine apoptotic cells by flow cytometry (Figures 4A,B respectively). Importantly, these clones previously demonstrated high binding to different citrullinated nuclear antigens by ELISA. There was no difference in binding to murine or human cells, or by apoptosis induction using different agents (dexamethosone, anti-Fas, or etoposide). In all cases, ACPA predominantly bound late apoptotic cells, with partial binding to early apoptotic cells, and no reactivity against healthy cells. Interestingly, co-incubation with the PAD-inhibitor Cl-amidine (Cl-A) during apoptosis did not affect the ACPA apoptotic cell binding, indicating either that the modified antigens are pre-formed, or that the binding is PAD-independent (Supplementary Figure 6).

Immunoblotting revealed that these ACPA bound strongest to proteins with molecular weights of ~17 and ~14 kDa in apoptotic cell lysates (Figure 4C). Immunoprecipitations of apoptotic Jurkat lysate with apoptotic cell-reactive ACPA (1325:01B09, 37CEPT2C04, 37CEPT1G09, 1325:05C06) showed capture of four proteins (13–14, 17–18, 18–20, and 36–37 kDa). Such binding was absent for control IgG or when using apoptotic cell-reactive ACPA together with lysate from untreated cells (Figure 4D). The clones 1325:01B09, 37CEPT2C04, 37CEPT1G09 showed the strongest binding, whereas 1325:05C06 showed considerably weaker co-precipitation. This clone also had less reactivity in the previous Western blot staining. Mass spectrometry analysis of the precipitated proteins identified histone 4, histone 2A, histone 2B/3, and histone 1, respectively. The 36–37 kDa band contained multiple proteins including actin. Furthermore, SDS-PAGE of the whole cell lysates before immunoprecipitations showed higher level of free histone protein in the apoptotic cell lysates than in the lysate from control treated cells (Supplementary Figure 7).

**Figure 5:**
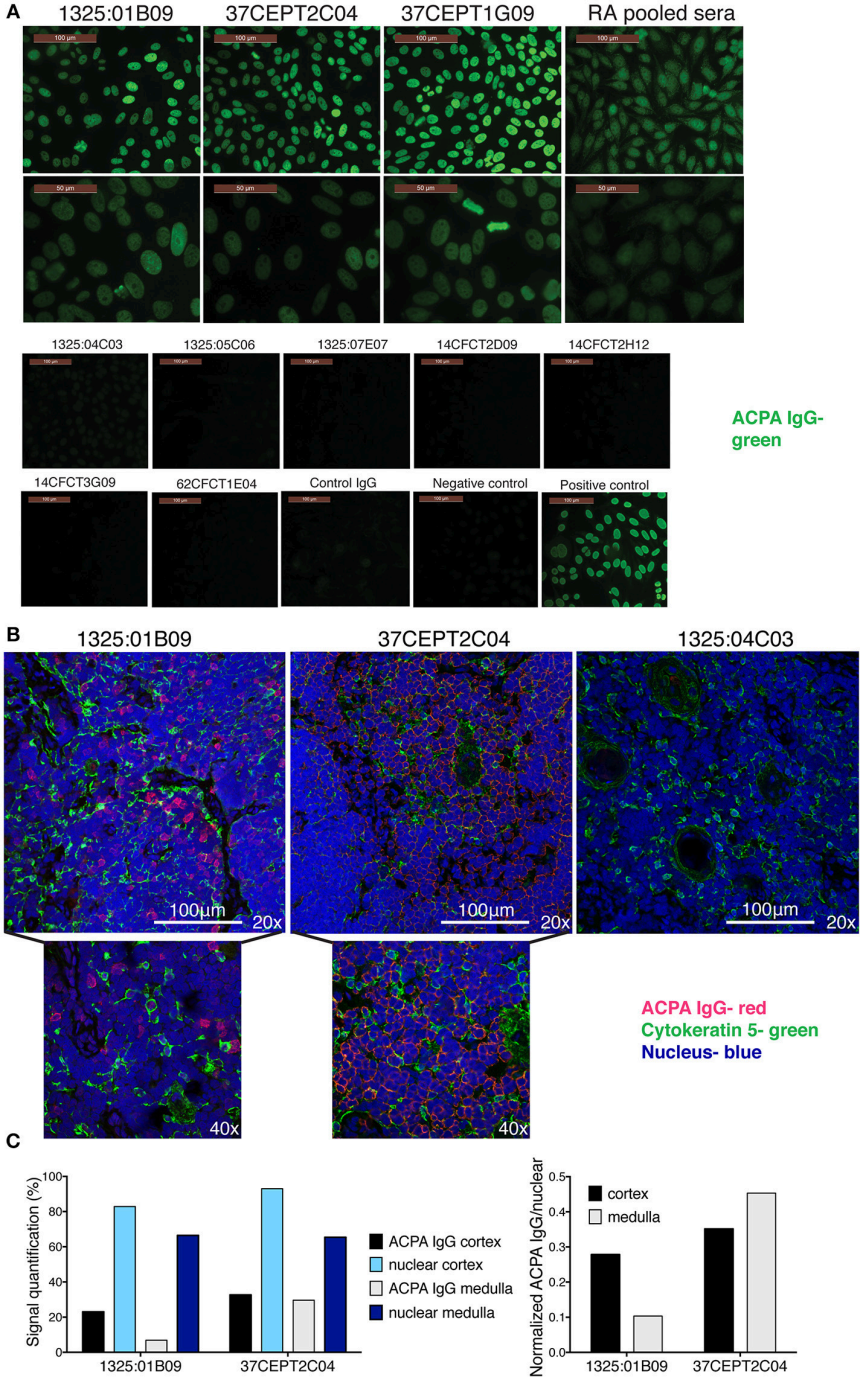
Anti-citrullinated histone ACPA produce nuclear staining pattern. **(A)** Anti-nuclear autoantibody reactivity (ANA) was determined for ACPA with HEp-2 slides (Inova Diagnostics). Antibody binding is visualized in green. **(B)** Monoclonal ACPA was evaluated for binding to human thymus tissue from children undergoing cardiac surgery by immunofluorescence staining. IgG binding is visualized in red, nuclei in blue, and medullary epithelial cells by cytokeratin five in green. ACPA hIgG1 mAbs were evaluated at 5 μg/ml. The figure shows representative images. **(C)**. Quantification of thymus staining of ACPA mAb IgG (1325:01B09 and 37CEPT2C04) and nuclear staining in medulla compared to cortex areas of the section (% area covered). Right panel show the cell density normalized quantification (ACPA IgG staining divided by nuclear staining). Details of the quantification can be found in Supplementary Figure 10.

To further characterize ACPA binding to nuclear antigen using conventional serologic methods, we screened the ACPA for anti-nuclear antibody (ANA) HEp-2 cell staining. Only a subset of the ACPA (1325:01B09, 37CEPT2C04, 37CEPT1G09) were positive for ANA staining (Figure 5A). They produced a nuclear staining pattern most closely similar to the dense fine speckled or AC-2 pattern according to the ICAP ANA nomenclature, with heterogeneity in brightness and distribution of speckles and the characteristic coarse speckled stained metaphase plate (Figure 5A). Notably, the ANA positive ACPA did not significantly bind to any purified native ANA autoantigens including histone, dsDNA, or DFS70 by line blot ANA profile screening. A weak binding to nucleosomes disappeared when mAb 37CEPT2C04 was diluted, while total IgG-binding remained; this was regarded as non-specific binding (Supplementary Figures 8B,C). Furthermore, the ACPA did not bind to dsDNA in ELISA using the methylated-BSA dsDNA capture protocol. If instead citrullinated histone coated wells were used, the ACPA-cit-His binding was completely blocked, and hence no binding was detected to histone-dsDNA complexes (Supplementary Figure 8A). We screen a limited number of ACPA-positive and ACPA-negative RA patient sera for HEp-2 ANA. The results show that there were no correlations between ANA patterns and neither ACPA positivity nor anti-histone reactivity (Supplementary Figure 9). Notably, the samples were screened at 1:40 dilution in concordance with the manufacturer's instructions. In our analysis, this potentially gives some false positivity compared to a more discriminating 1:160 dilution, but avoids that any binding capacity was missed (51).

In order to evaluate ACPA binding in the absence of inflammation, we took advantage of human thymus tissue which feature a dense population of T-cells naturally undergoing apoptosis, and should not express inflammation-related citrullination. Histology staining of fixed and permeabilized human thymus revealed that the tested ANA and apoptotic cell reactive ACPA (1325:01B09 and 37CEPT2C04) also bound intracellular targets (Figure 5B), which co-localized with nuclear staining. The clone 1325:01B09 showed a distinct binding pattern to nuclei with limited overlap with medullary epithelial staining, whereas the clone 37CEPT2C04 exhibited a wider reactivity yet bound only in certain cells within the tissue, which was quantifiable (Figure 5C, Supplementary Figure 10). Consequently, the ACPA do not bind to nuclei in all cells despite the nuclear reactivity, highlighting the specificity of binding. Flow cytometry confirmed that ACPA bound intracellularly rather than to the surface of human thymocytes (Supplementary Figure 10). Together, the data shows that among the ACPA investigated here a subset can bind to nuclear structures and apoptotic cells, and that the reactivity seems to be primarily directed toward histones.

### ACPA mAbs and Apoptotic Cells Increase Citrulline Responses *in vivo* but the Effect Is Not Dependent on Binding to Nuclear Antigens

**Figure 6:**
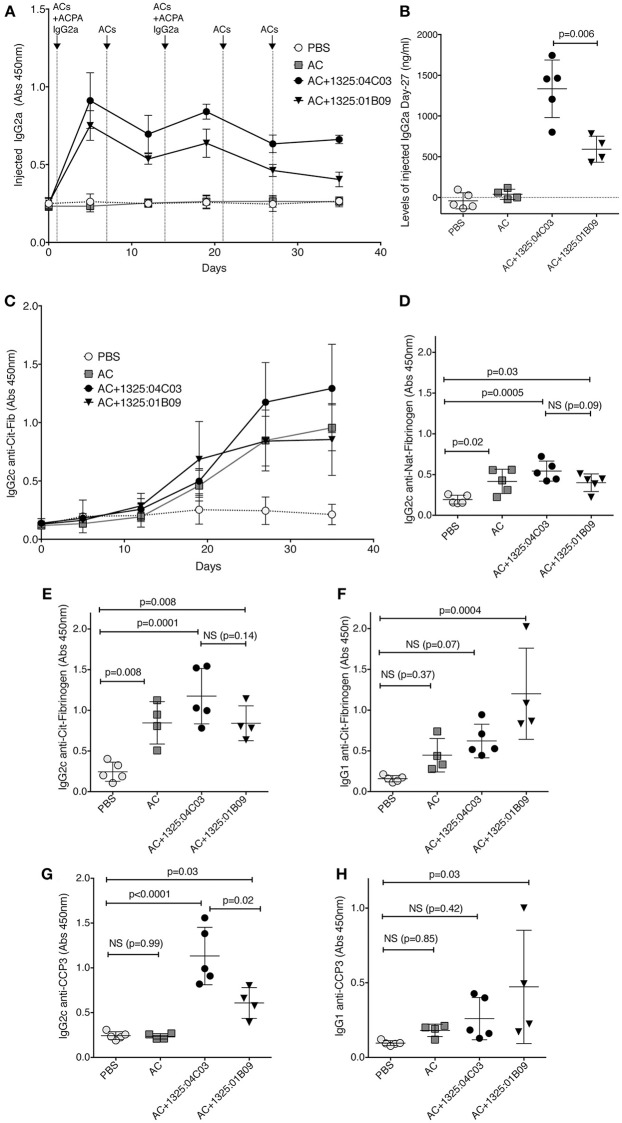
ACPA mAbs in an *in vivo* model of apoptotic cell induced autoimmunity increase citrulline responses. C57BL/6 mice were injected i.v. with 10^7^ apoptotic thymocytes at four occasion, one week apart. One milligrams of murine chimeric IgG2a ACPA mAbs were given in combination with the first and third injection. The figure depicts antibody serum levels determined by sandwich ELISA. Subclass specific detection antibodies (Southern Biotech) were used for separating injected IgG2a from endogenously expressed IgG2c and IgG1. **(A)** Serum levels of injected ACPA monoclonal mIgG2a antibodies were detected with subclass specific secondary antibody. **(B)** Serum levels of injected ACPA murine IgG2a on day 27 after the start of the experiment. Unspecific reactivity of the secondary antibody was removed by subtraction of values for pre-injection mice. **(C)** Induced murine IgG2c to citrullinated human fibrinogen. **(D,E)** Induced mIgG2c to native and citrullinated fibrinogen at day 27. **(F)** Induced mIgG1 to citrullinated fibrinogen at day 27. **(G,H)** Induced serum IgG2c or IgG1 anti-CCP at day 27 detected with a subclass specific detections reagent using the commercial CCP3 coated wells (QUANTA Lite CCP3 IgG, Inova Diagnostics). Groups contained 4–5 mice. *P*-values are shown from ANOVA analysis with correction for multiple comparisons.

To further query the functional relevance of autoantibody binding to apoptotic cells *in vivo*, we investigated two monoclonal ACPA, one with anti-nuclear binding (1325:01B09) and one without (1325:04C03), in a murine model of apoptotic cell-induced autoreactivity (50, 52, 53). By utilizing validated subclass specific detection reagents (Supplementary Figure 11), injected chimeric murine IgG2a could be separated from endogenous IgG2c and IgG1 responses. Some immunogenicity of the chimeric mIgG2a could be detected at day 35 after injection but did not affect the overall analysis (Supplementary Figure 12). We found that circulating levels of 1325:04C03 were significantly higher than for 1325:01B09 (*p* = 0.006, Figures 6A,B). Intriguingly, trends were observed for an elevated IgG2c immune response to citrullinated fibrinogen (Figure 6) as well as to nuclear proteins (Supplementary Figure 13) in mice treated with 1325:04C03 but not with 1325:01B09, when compared to the injection of ACs alone. No reactivity to citrullinated histone or vimentin could be detected in serum from the mice (Supplementary Figure 14). Notably, the clone 1325:04C03 does not itself react with citrullinated fibrinogen, excluding any risk of the injected mAb giving false positivity in the ELISA, and the maintained distinct specificities of the two injected mAbs could also be clearly seen when screening cit-vimentin vs. cit-fibrinogen IgG2a in the serum of treated mice (Supplementary Figure 11). Treatment with the clone 1325:01B09 in combination with apoptotic cells instead increase an IgG1 response, although there was a large variation between individual mice. Furthermore, we could observe a significant increase in IgG2c reactivity to citrullinated peptides measured by anti-CCP3 reactivity in the 1325:04C03 injected compared to 1325:01B09 injected mice (Figure 6D), which could not solely be explained by any potential cross-reactivity of the subclass-specific reagent. In 1325:01B09 treated mice, we could also detect increased IgG1 anti-CCP3. While there was a weak correlation between circulating levels of the injected IgG2a and IgG2c anti-CCP3 responses, no such correlation could be seen for IgG1 anti-CCP3 (Supplementary Figure 15). The response was specific since no CCP3 reactivity was seen in mice eliciting other unrelated immune responses such as mice immunized with ovalbumin (Supplementary Figure 16).

In conclusion, free ACPA may increase the immune response to citrullinated antigens but we cannot detect any clear indication of either enhanced or decreased immunological effect by the anti-apoptotic cells reactive ACPA compared to non-reactive ACPA in apoptotic cell injected mice. This may be consistent with the fact that the anti-apoptotic cell ACPA mAbs did not mediate phagocytosis of apoptotic cells *in vitro* (data not shown). Yet, our data suggest that circulating ACPA in combination with apoptotic cells may mediate induction of autoreactivity to citrullinated epitopes *in vivo*, while apoptotic cells alone induce ANA and possibly a more polyreactive response to full-length citrullinated protein.

### ACPA Monoclonals With Anti-nuclear Reactivity Display PAD-Independent Binding to Neutrophils and NETs

We next explored how anti-nuclear ACPA reactivities were associated with binding to activated neutrophils and NETs using two different cell systems; primary human neutrophils and a murine neutrophil system based on differentiation of the estrogen-dependent myeloid stem cell line ECoM-G (46). Stimulation with PMA for human cells and ionomycin for murine cells induced *in vitro* NETosis reactions that could be evaluated by microscopy fluorescent staining of nucleic acid.

**Figure 7:**
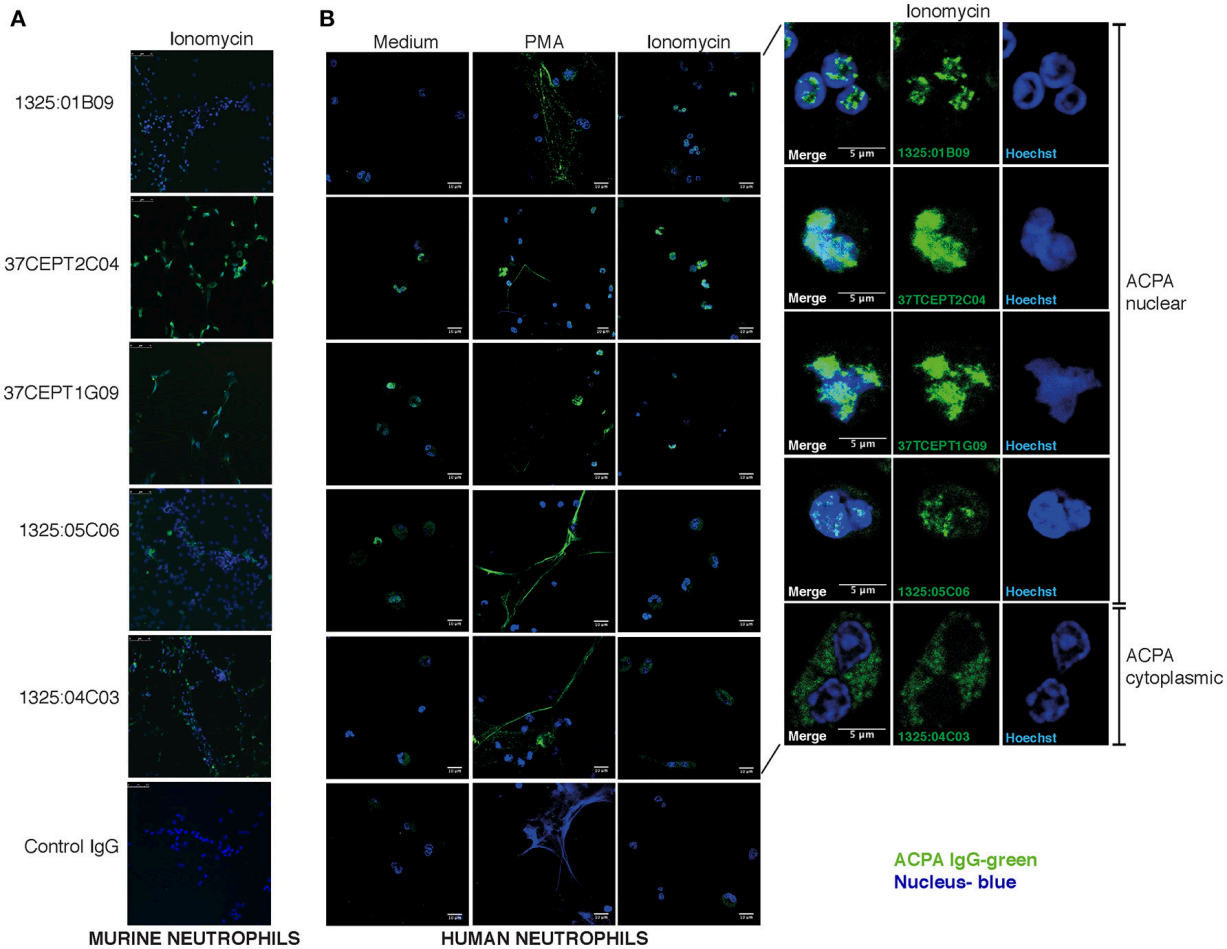
ACPA subgroups can be defined by different binding patterns to neutrophils and NETs. Immunofluorescence staining of ACPA mAb binding to neutrophils and NETs. **(A)** Ionomycin (4 h, 1 μM) activated murine ECoM-G differentiated neutrophil cells. **(B)** Primary human neutrophils that were control stimulated (medium) or stimulated with PMA (4 h, 50 nM) or ionomycin (4 h, 1 μM). ACPA mAb binding to permeabilized and fixed cells was evaluated at 5 μg/ml using hIgG1 versions for murine cell binding and murine chimeric IgG2a mAbs for human cell experiments. ACPA IgG binding is visualized in green and nucleus in blue. The figure shows representative images from three repeated experiments.

**Figure 8:**
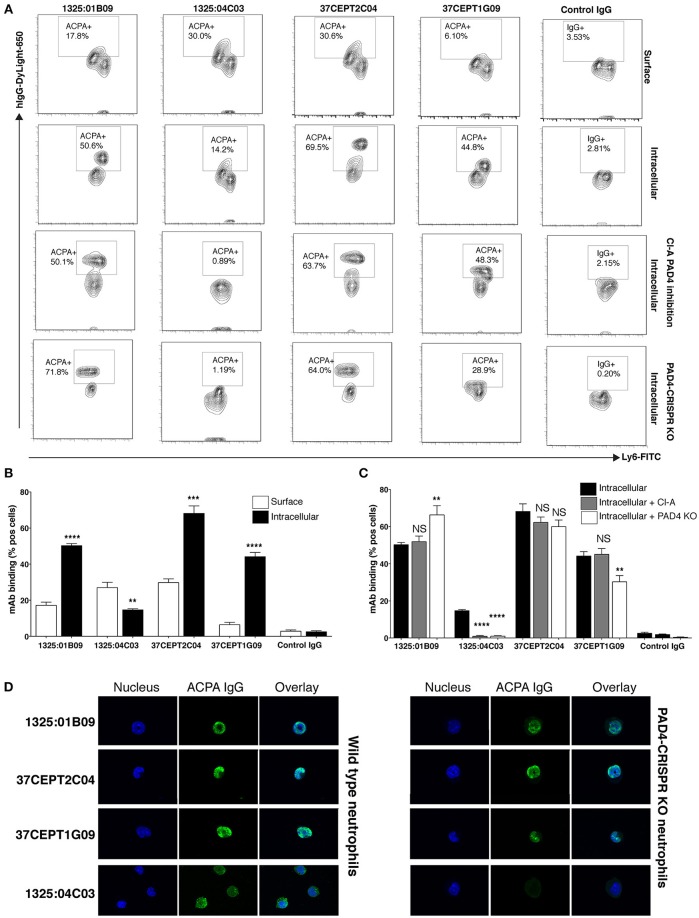
Anti-nuclear ACPA binding to neutrophils is not dependent on PAD4. Flow cytometry binding of human ACPA monoclonal antibodies (10 μg/ml DyLight-650 hIgG1) to neutrophil differentiated murine ECoM-G cells after ionomycin stimulation (4 h, 1 μM). Binding was evaluated with and without permeabilization using the Foxp3 kit (eBioscience) showing surface staining compared to intracellular staining **(A,B)**. ACPA binding was also evaluated in ECoM-G cells with differentiated in the presence of Cl-Amidine (Cl-A,100 μM) for 5 days and to CRISPR-Cas9 PAD4 KO cells **(A,C)**. **(A,B)** summarize the flow cytometry % binding (Mean and SD) for triplicate conditions in one representative experiment. The figure shows representative images from three repeated experiments. **p < 0.01–0.001, ***p < 0.001–0.0001, ****p < 0.0001 derived from student *t*-test comparing intracellular binding to surface binding **(A)** or binding to cells without treatment **(B)**. **(D)** Immunofluorescence staining of ACPA mAb binding to ionomycin stimulation of wild type ECoM-G neutrophils compared to CRISPR-Cas9 PAD4 KO cells. The figure shows represented images of stimulated cells in early NETosis.

The ACPA clones (1325:01B09, 37CEPT1G09, 37CEPT2C04, 1325:05C06) showed strong binding to PMA-stimulated human neutrophils exhibiting NETosis, as well as ionomycin stimulated cells (Figure 7). During NETosis, these ACPA mAbs co-localized with both nuclear targets and NET components. Notably, the clone 1325:05C06, which featured apoptotic cells binding but no HEp-2 ANA reactivity, was positive for NET binding (Figure 7). The 1325:04C03 clone, which lacked nuclear reactivity, instead showed a cytoplasmic binding pattern in activated neutrophils together with a strong NET binding, and did not bind to unstimulated cells. The binding of ACPA mAbs to human neutrophil NETs were confirmed in the ionomycin stimulated ECoM-G murine cells (Figure 7). Opposite to the human system, ionomycin induced higher ACPA binding than PMA stimulation. Importantly, flow cytometry analysis of ACPA binding to murine neutrophils was able to confirm and quantify the different binding reactivities from the human neutrophil study (Figure 8). For example, the clone 1325:04C03 displayed more intracellular than surface binding when comparing protocols with or without permeabilization. Notably, while flow cytometry can to some extent be used for detecting NET-products intracellularly (with permeabilization) as well as exposed on the cell surface (without permeabilization) during activation, it may not detect neutrophils fully in NETosis as these may be too sticky for the analysis. The ACPA clones 1325:01B09, 37CEPT2C04, and 37CEPT1G09 all possessed high intracellular binding consistent with nuclear-binding (Figure 8).

In addition, the ACPA mAbs were evaluated for reactivity to primary murine bone marrow cells (Supplementary Figure 17). Intriguingly, the cytoplasmic neutrophil reactive ACPA clone 1325:04C03 displayed significantly higher binding to differentiated (linage positive) cells while the other clones (1325:01B09, 37CEPT2C04, and 37CEPT1G09) exhibited higher binding to precursor cells (Supplementary Figure 17). In the ECoM-G differentiated neutrophils, we could also distinguish two cell subsets based on Ly6G expression with different ACPA-binding for some mAbs, yet these results may not be easily translated into the situation in primary cells.

As PAD4-mediated citrullination has been reported to be a crucial step in NETosis (16), we questioned whether PAD4 contributed to the observed differences in ACPA mAb binding to activated neutrophils and NETs. Notably, in these experiments we utilized Ca-dependent ionomycin induced NETosis, a protocol that is considered to induce high level of citrullination. As shown in Figure 8A, the addition of a high dose of PAD4 inhibitor (Cl-amidine) during ECoM-G stem cell-to-neutrophil differentiation significantly reduced binding of 1325:04C03 after ionomycin stimulation, whilst binding of the nuclear-reactive ACPA were unaffected. To further investigate PAD4-dependence, we applied a CRISPR-Cas 9 approach to generate an ECoM-G PAD4 knock-out cell line, whereby the absence of expressed PAD4 protein was confirmed by Western blot (Supplementary Figure 18). Whilst neutrophil differentiation was unaffected in these cells, they displayed reduced ability for NETosis. Despite this, the ANA reactive ACPA mAbs (with a citrullinated histone reactivity profile) maintained comparable reactivity to the PAD4-KO neutrophils (Figure 8A,C,D). The binding of the clone 37CEPT1G09 was reduced, whereas the binding for 1325:01B09 and 37CEPT2C04 mAbs were similar or even increased. However, for the cytoplasmic reactive ACPA 1325:04C03, the binding was significantly lower or absent in the PAD4 KO cells. To further evaluate the independence of PADs and citrullination, we studied binding to ionomycin-stimulated bone marrow neutrophils from PAD2 and PAD4 KO mice (54). The nuclear-reactive mAbs showed equally strong binding to the cells from both PAD2 and PAD4 KO mice (Supplementary Figure 19). Yet, we could confirm a reduction in citrullination in both ECoM-G PAD4 CRISPR-KO and cells from the PAD4 KO mouse using a chemical citrulline reactive fluorescent probe, especially in proteins with equivalent in size to histones (Supplementary Figure 20).

### The Nuclear Reactive Subset of ACPA Cross-React With Acetylated Epitopes in Histones

**Figure 9:**
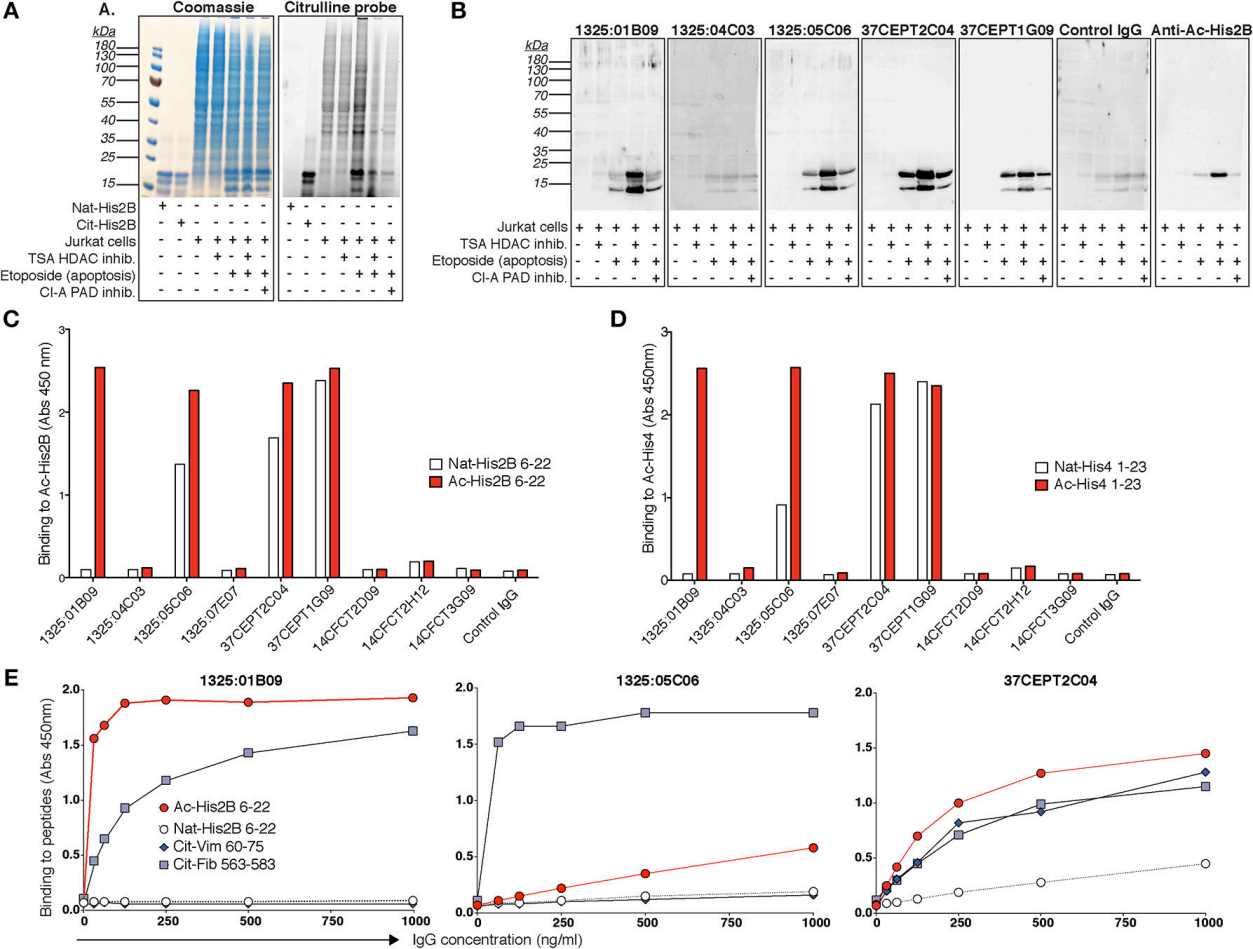
Citrulline-independent ACPA mAb binding to histones in NETs and nuclei can be explained by binding to acetylated histone epitopes. **(A)** Citrullination was evaluated using a rhodamine-based chemical probe in Jurkat cells treated with the 100 ng/ml histone deacetylase (HDAC) inhibitor trichostatin A (TSA) or 20 μM of the PAD inhibitor Cl-amidine (Cl-A) 3 h before induction of apoptosis with 25 μM etoposide overnight. HDAC inhibition leads to increased acetylation but no significant change in citrullination. The right panel shows the cell lysates separated by SDS-PAGE and stained with the citrullination probe and the left panel shows the same gel stained with Coomassie. **(B)** Western blot binding of ACPA hIgG1 monoclonal antibodies (5 μg/ml) to acetylated histones in treated Jurkat cell lysates detected with rabbit anti-human IgG HRP. Acetylated histone 2B was detected as a control using a specific antibody (Cell Signaling). Reactivities of the ACPA IgG mAbs (5 μg/ml) to biotinylated acetylated peptides from histone 2B (Ac-His2B 6–22) **(C)** and histone 4 (Ac-His4 1–23) **(D)** were confirmed with ELISA. **(E)** ELISA titration of three acetylated-histone positive ACPA mAbs to acetylated histone 2B peptide (Ac-His2B 6–22), compared to the native lysine peptide and two citrullinated peptides from vimentin (cit-Vim 60–75) and fibrinogen alpha chain (cit-Fib 563–583), at the indicated IgG concentrations. The figure shows representative data from three repeated experiments.

**Figure 10:**
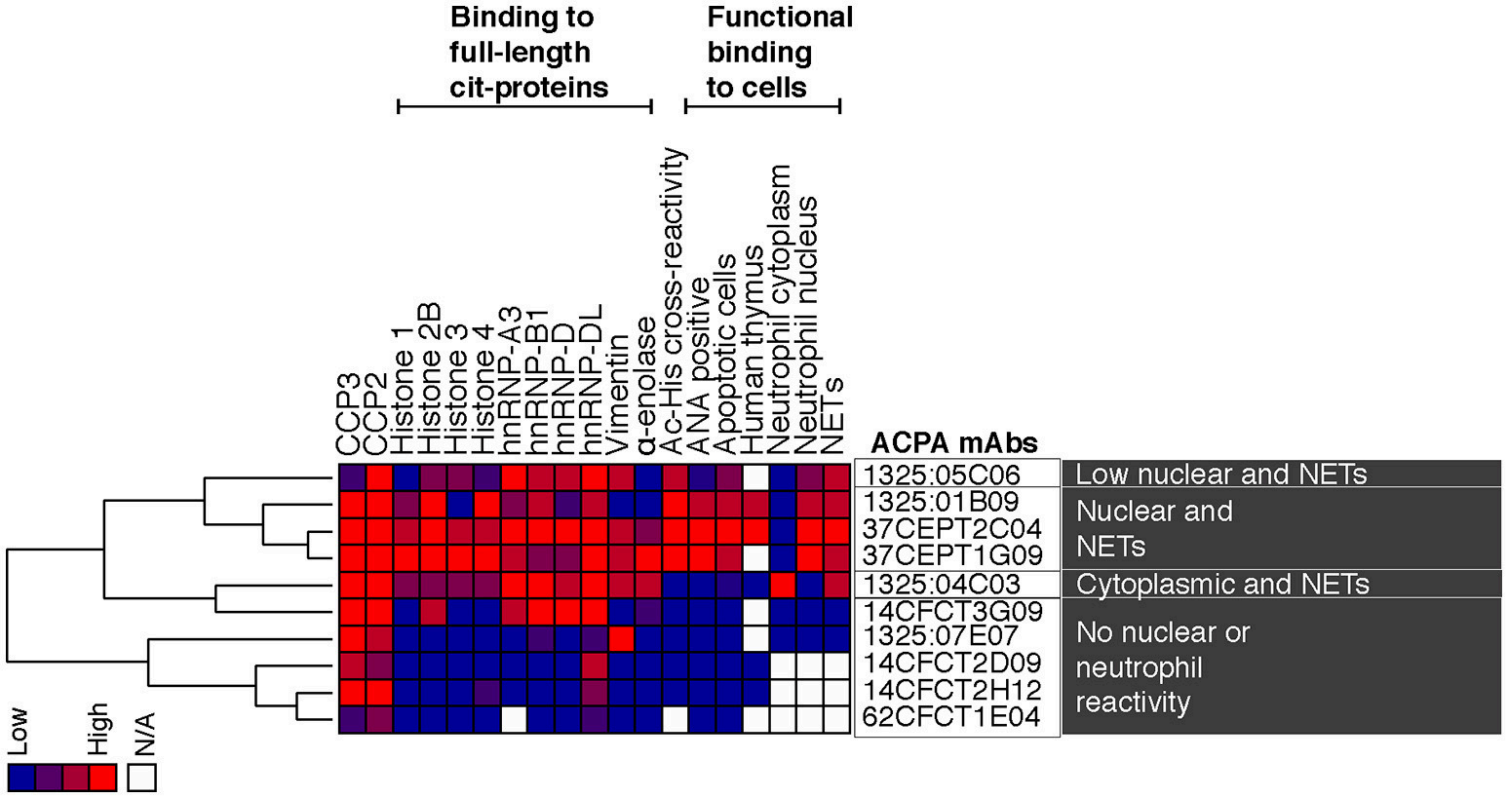
Summary of reactivity patterns for evaluated monoclonal ACPA. The figure summarizes ELISA binding reactivities for the investigated RA-derived human ACPA monoclonal antibodies together with results from cell interaction studies. The investigated single cell isolated ACPA monoclonal antibodies were derived from synovial plasma cells (1325:01B09, 1325:04C03, 1325:05C06, 1326:07E07) or antigen-tetramer captured circulating memory B cells (37CEPT1G09, 37CEPT2C04, 14CFCT2D09, 14CFCT2H12, 14CFCT3G09, 62CFCT1E04) from four different CCP-positive RA patients (RA1325, RA37, RA14, and RA62) (5, 10). The antibody binding was normalized 0–4 and the monoclonal clones were analyzed by hierarchical clustering with complete linkage using Cluster 3.0 and visualized by Java Treeview. The heat-map shows high reactivity in red, intermediate in purple and negative results in blue.

In the light of the PAD-KO experiments, we wondered if the ACPA would cross-react to other PAD-independent post-translational modifications in the nucleus. By using the fluorescent citrulline probe we confirmed that citrullinated-histones are increased in apoptotic cell lysates and that citrullination can be inhibited by the PAD inhibitor Cl-A (Figure 9). Yet, Cl-A did not affect the ACPA-histone binding in Western blot. Moreover, detailed mass spectrometry analysis from immunoprecipitation experiments revealed that anti-nuclear ACPA captured acetylated histone proteins rather than citrullinated histones, and acetyl-lysine (Ac) peptides from acetylated His2B, His3.1, and His4 were identified (Supplementary Tables 2, 3) whereas no citrullinated peptides were detected. Furthermore, in Western blot of treated Jurkat cells, we detected significantly increased mAb binding using the broad histone deacetylase (HDAC) inhibitor trichostatin A (TSA) that gives elevated acetylation (Figure 9). The citrulline probe confirmed that citrullination was not significantly affected by HDAC inhibition and anti-Ac-His2B detection showed acetylation of histone 2B (Figure 9). The ACPA mAbs demonstrated consistent binding to two acetylated bands after HDAC inhibition with MW consistent with different histone isoforms (e.g., His2B/3 and His4). We next used ELISA to determine binding to synthetic acetylated peptides covering know histone acetylation residues (Ac-His2B K12; Ac-His4 K5, K8, K12, K16), and we could confirm that only the ACPA with ANA, apoptotic cell, and neutrophil nuclear patterns, bound to acetylated histone peptides (Figure 9). The ELISA reactivity to acetylated peptides were similar in strength compared to the cit-peptides Cit-Vim60-75 and Cit-Fibα563-583 that represent well-characterized ACPA cit-targets (Figure 9, Supplementary Figure 21). While the mAb 1325:01B09 displayed no detectable binding to any native peptides, 37CEPT1G09 and 37CEPT2C04, and to minor extent 1325:05C06 showed some reactivity also to native lysine-containing His-peptides at high IgG concentration, however this was to large extent diminished when titrating to lower concentrations. The acetylation-reactivity was also present in polyclonal anti-CCP2 purified ACPA pools from RA patients (Supplementary Figure 22). Furthermore, germline converted versions of the clones 1325:05C06 and 1325:01B09 (10) did not bind acetylated peptides (Supplementary Figure 21). Hence, this reactivity, similar to what has been seen previously for citrullinated peptides (10), are generated by considerable degree of somatic hypermutation and affinity maturation of B cells in the germinal center. In summary, our results reveal a distinct ACPA subset based on monoclonal antibody reactivity patterns to activated neutrophils and NETs, and highlight a surprising difference in dependence for PAD2/4-citrullination and reactivity to acetylated histones (summarized in Figure 10).

## Discussion

Important new findings presented in this study include the following observations: There is a major heterogeneity among monoclonal ACPA generated from B cells from RA patients; We find that some, but not all, of these monoclonal ACPA show strong anti-nuclear binding patterns including binding to apoptotic cells; These anti-nuclear ACPA also react with activated neutrophils and NETs and the binding of these antibodies is largely independent on PAD-expression and is mediated by acetylation. This is not the case for another ACPA subset that display a citrulline-dependent NET and neutrophil cytoplasmic binding. Taken together, our studies demonstrate how detailed investigation of ACPA reactivity using patient-derived monoclonal antibodies, rather than serum polyclonal antibodies, can reveal functional differences of potential pathogenic significance between ACPA subsets. Mechanistically, this may also suggest potential differences in the origin of these subsets, including the B cell population recruited and the nature of the antigen.

Despite the emerging data on a pathogenic role of ACPA in RA (2–11, 55, 56), there is still limited knowledge concerning how the detailed specificities of different ACPA relate to functionality or to targeting of different effector cells. Protein-modification can occur in many different situations, and we don't fully understand which processes may initiate break of tolerance and drive the continuous anti-citrulline response in RA. Synovial hypercitrullination has been postulated to be unique for RA pathogenesis (57). Yet, this process is likely more associated with chronic disease than physiological conditions or pre-disease autoimmunity. However, modified antigens generated by cell death and NETosis could instead be hypothesized to play a role in initiating or amplifying autoreactivity. While ACPA-NET interactions have previously been reported (22), there has been little focus on apoptosis in the context of RA to date. Autoreactivity to components of dead cells and nuclear antigens may reflect a general lack-of-tolerance toward modified molecules generated during apoptosis and such binding has previously been described for the healthy immune system with polyreactive antibodies (58) and natural IgM (59).

We identified histones as a target for ACPA in apoptotic cells and initially focused on citrullinated histone 2B. Citrullinated histones have previously been shown to be immunostimulatory by themselves, and *in vitro* formed citrullinated histone-IgG immune complexes stimulated macrophages in a mechanism proposed to occur via TLR-Fcγ receptor cross-talk (8, 11). However, in our assay, the effect was solely dependent on antigen-binding and FcγR activation. Importantly, we demonstrate a significant heterogeneity between different monoclonal ACPA with respect to this type of immunostimulation, as we find that one ACPA clone (1325:01B09) specifically induced proinflammatory cytokine production. Importantly, the finding that ACPA-citrullinated histone immune complexes drive IL-8 production connects these antibodies to this chemokine in early phases of RA. Mechanistically, IL-8 has been shown to be central to causing bone loss and pain behavior in models of early RA (4, 6). The degree of ACPA citrullinated peptide cross-reactivity in immune complexes obtained *in vivo* from RA synovial fluid was recently shown to be associated with the extent of joint destruction and measures of inflammation (56).

An interesting finding highlighted here was that some monoclonal ACPA displayed ANA reactivity. Although 20% of RA patients have been reported to have serum ANA reactivity, this has not been associated with ACPA status (60). Patient-derived monoclonal ACPA with citrullinated histone and apoptotic cell reactivity were here shown to display ANA HEp-2-staining consistent with chromatin-related patterns. This binding had more similarity to the dense fine speckled or AC-2 pattern according to the ICAP nomenclature than to the regular anti-native histone pattern (44). Additionally, the ANA-positive ACPA subset exhibited strong binding to nuclear structures in human thymus. The differential binding to the nuclei of distinct cells, particularly with the clone 1325:01B09, may suggest diversity in nuclear protein modification, potentially in association with differentiation or proliferation. Importantly, the ACPA mAbs did not display any significant binding to purified native histones, nucleosomes or dsDNA complexes in control ANA-antigen assays, and did not display any similarities with reported lupus-associated or murine anti-histone or anti-DNA reactivities (61, 62).

Increased intracellular calcium levels during apoptosis may induce increased PAD enzyme activity (63) and if citrullination of histones occurs it could potentially contribute to histone remodeling. Although histone citrullination has been hypothesized to be a prominent feature during apoptosis and autophagy, it had not been investigated previously in this context. Citrullination in neutrophils has been reported to not be increased during apoptosis (64), yet, our studies using the chemical rhodamine citrulline-probe clearly indicate an increase of citrullinated histone protein during apoptosis in Jurkat cells.

Increased amounts of histone proteins within the soluble phase of cell lysates were also detected, consistent with a previous finding that histones are released from nucleosome complexes during apoptosis (65) and are more accessible for captured by anti-histone antibodies (66). Besides increased PAD activity, apoptosis may also lead to increased translocation and exposure of modified antigens on the cell surface, or breakdown of membrane structures leading to higher accessibility to intracellular materials for antibody binding. Therefore, it can be hard to discriminate between increased accessibility or increased expression in apoptotic cell assays. Yet, our mAb-binding results were consistent between flow cytometry and western blot analysis of whole cell lysate, supporting that the antigen level is indeed elevated during apoptosis. Citrullination of vimentin has been suggested to be important during apoptosis, contributing to morphological changes (67). However, no binding to apoptotic cells was seen for the investigated ACPA clones with documented high binding to citrullinated vimentin (e.g., 1325:04C03). Hence, our results demonstrate that citrullination of vimentin is limited during the different apoptosis pathways included in our investigation or is not accessible for detection (i.e., anti-Fas, etoposide, or dexamethasone, in T-cells).

Importantly, nuclear ACPA mAb binding to apoptotic cells could only partly be explained by reactivity to elevated cit-histones. We therefore concluded that the ACPA may instead bind to another apoptosis-associated histone post-translational modification with similar chemical properties. Histone acetylation is a neutrally charged lysine modification that is associated with chromatin relaxation and gene activation, but is also increased in apoptosis (68). Indeed, the ACPA subset with anti-apoptotic cell reactivity was found to have strong binding to acetylated histones within lysates of stimulated cells and direct reactivity to acetylated histone 2B and 4 peptides. This Ac-histone reactivity fully explained the PAD- and citrulline-independent binding of certain ACPA to nuclear structures.

Cross-reactivity of ACPA mAbs to acetylated histones peptides has not been previously reported. Polyclonal RA antibodies have been shown to contain reactivity to acetylated residues using a synthetic, not naturally occurring, construct based on a vimentin peptide whereby the arginine/citrulline residue was exchanged for acetyl-lysine (69, 70). Our ELISA results with ACPA mAbs show that only the antibodies recognizing a modified consensus motif present in the vimentin peptide have the potential to bind these carb- and acetyl- synthetic vimentin peptides (10).

In the current study, the acetyl-histone peptides are containing glycine in the −1 position and therefore cross-reactive mAbs are only found among the mAbs binding to a Cit-Gly dominating consensus motif. The clone 1325:01B09 has previously been determined to also cross-react to carbamylated (homocitrullinated, carb) peptides displaying the Carb-Gly consensus motif (10). Identified carb-peptides among 49,211 peptides (Roche NimbleGen microarray) included peptides from histone 2B and histone 4 covering the known lysine acetylation sites that we included in the Ac-peptides in the present study (His2B K12; His4 K6, K8, K12). Thus, some overlap in recognition of carbamylating and acetylation may be expected. In conclusion, cross-reactivity of ACPA to acetylated histones represents a new autoreactivity in RA which is observed in a subset of ACPA autoantibodies. The acetylation reactivity was equal or stronger than citrulline reactivity and potentially representing a more dominant binding for these clones *in vivo*. Owing to the extensive modified protein cross-reactivities, perhaps these and other ACPA should rather be considered as AMPA.

In a previous report, proteomic analysis of activated neutrophils identified citrullinated Histone 2B as a target of anti-modified citrulline antibodies, which possessed the capacity to induce macrophage cytokine production and propagated neutrophil activation (8). PAD4-dependent NETosis has previously been shown to occur under certain forms of stimulation (16, 71, 72), and we confirm a reduction in Ca-dependent NETosis in the CRISPR-PAD4 KO cells. NET citrullinated histone 4 has additionally been to shown to be a significant target of serum antibodies and ACPA reactivity to cit-His4 peptides is commonly detected in RA patients, which correlates with anti-CCP2 levels (73). Furthermore, monoclonal ACPA binding capacity to NETs has been reported previously (22). Yet, here we demonstrate the nuclear-reactive ACPA subset interact strongly with activated neutrophils and NETs in a PAD-independent manner. The anti-nuclear ACPA maintained strong binding to the PAD4 KO cells as well as cells from PAD2 KO mice whilst binding was completely lost for the anti-cytoplasmic ACPA (1325:04C03) that displayed PAD4-dependent binding. Hence, we conclude that the anti-nuclear subset of ACPA bind strongly to NETs through mechanism that are likely involving acetylated histones, similarly to what was seen in the apoptotic cells. Acetylation of histones has been demonstrated in NETosis and have been reported to be immunostimulatory as well as a suggested autoantibody target in SLE (74–76), but has not yet been discussed in the context of RA. The difference in the studied ACPA mAb binding patterns further emphasizes the appearance of distinct ACPA subsets with potentially different functionality. It is important to state that the murine *in vitro* cell system utilized Ca-dependent NETosis, which may not fully mimic NETosis *in vivo* or NETosis induced by microbial stimuli, and gives a higher level of citrullination, although not to the same extent as leukotoxic-induced hypercitrullination (77). However, our starting point was the investigation of mAb-binding and interestingly we observed differences in stimulation of human blood neutrophils compared to murine bone marrow neutrophils. In the human system, we had equal binding of the different subtypes of ACPA to PMA compared to ionomycin stimulated cells while for murine cells we observed higher binding in ionomycin stimulated cells. Most importantly for the current study, the nuclear and cytoplasmic binding patterns of the different ACPA clones, driven either by acetylation or citrullination, respectively, remained consistent between neutrophil origin.

The fact that monoclonal ACPA clones with different fine-specificity patterns may mediate different functionality related to different symptoms of RA, is to some extent supported by our previous studies. For example, the 1325:04C03 clone has been demonstrated to have pathogenic properties by enhancing osteoclastogenesis and mediate osteoclast-driven IL-8 production, whereas the investigated monoclonal antibodies from the nuclear reactive subset did not possess such effects (10). We have also observed that certain monoclonal ACPA from the nuclear reactive subset enhance endotoxin-induced arthritis *in vivo* (5).

In conclusion, we demonstrate that certain ACPA specifically bind to nuclear and NETosis associated antigens. One type of ACPA reactivity to nuclear antigens in neutrophils and NETs is PAD-independent and driven by acetylation, whilst another subset of ACPA reactivity, with a cytoplasmic and peri-nuclear pattern, is dependent on PAD4 expression and citrullination. The studied monoclonal antibodies are bona fide highly mutated anti-citrulline antibodies without any unspecific polyreactivity and possess identical or similar properties to monoclonal antibodies published by other research groups (11, 78). They display substantial modified protein cross-reactivity due to the fact that they recognize distinct consensus peptide motifs, present in numerous full-length proteins, and our data now reveals that this motif can include different neutrally charged post-translational modifications including citrulline, homo-citrulline, and acetylation. This substantially expands our understanding of AMPA and emphasizes the considerable overlap between different reactivities to post-translational modifications. It also evokes important questions about which protein modifications primarily drive the initiation of autoimmunity, as well as the continuation of pathogenic responses in RA. Importantly, the current study emphasizes that ACPA can, and probably should, be divided into different subsets based on their cell-reactivity and modification cross-reactivity patterns. We propose that this subdivision of ACPA, now made possible with the availability of an increasing number of well-characterized monoclonal ACPA from RA patients, will lead to a better understanding of the pathogenic roles of different ACPA in relation to mechanisms and symptoms in RA, thereby enabling better interventions in this disease.

## Author Contributions

All authors were involved in drafting the article or revising it critically for important intellectual content, and all authors approved the final version to be published. KL, GW, CG, VM, FW, and LK designed the studies and interpreted the data. JS, PT, DM, and VM designed and performed single cells isolation of monoclonal ACPA and initial characterization of the antibodies. KL, GW, KC, DZ, CG, and PS performed ACPA binding experiments and analyzed data. BM, PS, and KS worked on expression and validation of recombinant autoantigen. RS produced and validated monoclonal antibodies. DZ performed PMBC assays, serological patient screenings, and analyzed clinical data. MJK provided materials and input on PAD KO cell experiments and interpretation of data. JR provided interpretation of ANA reactivity patterns and input on immune complex methodology. EO performed mass spectrometry experiments and analysis. MG and MCIK designed and performed *in vivo* murine studies. CL and OE worked on studies of ACPA binding to human thymus. KL and CG wrote the first manuscript draft. All authors participated in discussions and the finalizing of the manuscript.

### Conflict of Interest Statement

The authors declare that the research was conducted in the absence of any commercial or financial relationships that could be construed as a potential conflict of interest.
