# Supplementary material for: Differential ACPA Binding to Nuclear Antigens Reveals a PAD-Independent Pathway and a Distinct Subset of Acetylation Cross-Reactive Autoantibodies in Rheumatoid Arthritis
Source: Front Immunol. 2019 Jan 4;9:3033. doi: 10.3389/fimmu.2018.03033 (PMC6328449; doi:10.3389/fimmu.2018.03033)
Supplement: Supplementary file 1 [file Data_Sheet_1.PDF]

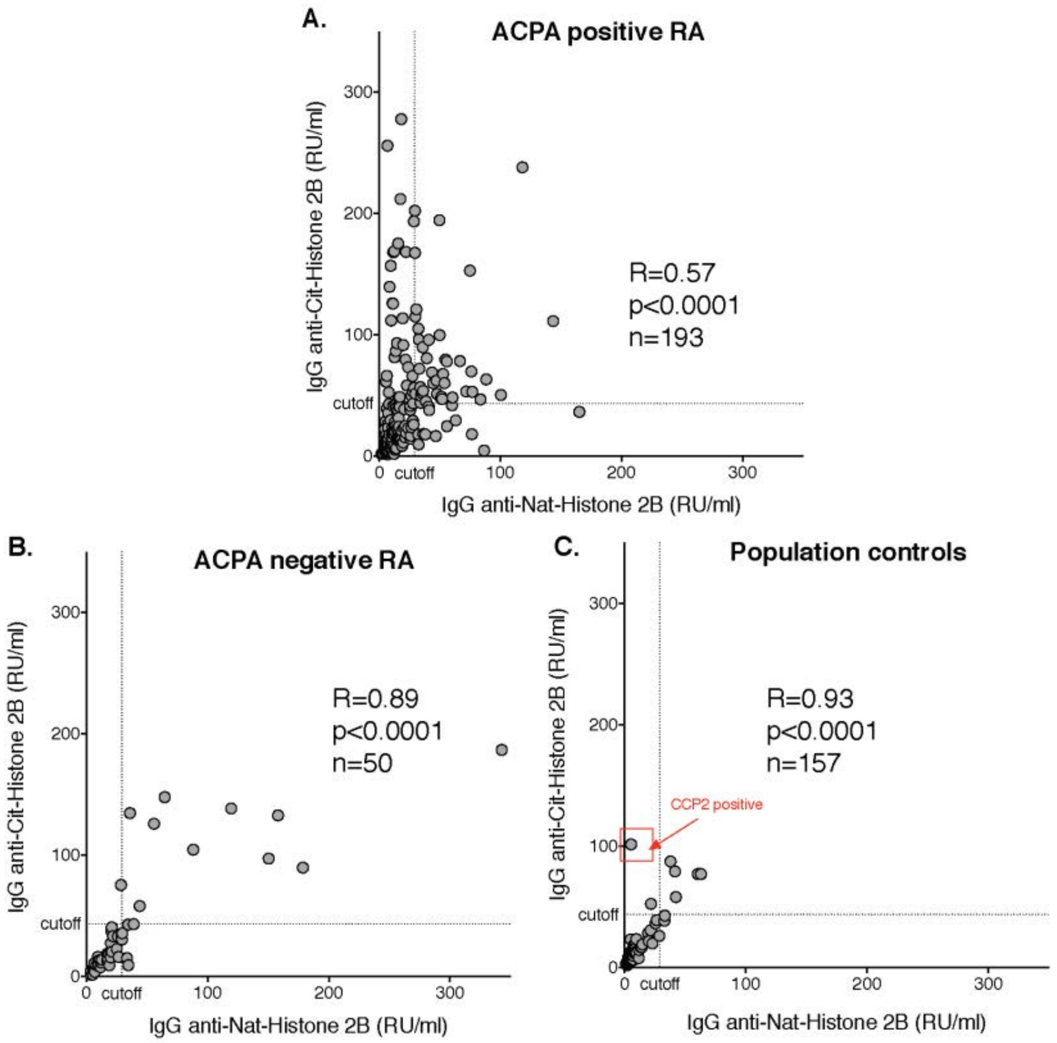

Supplemental Figure 1. Correlation between native and citrullinated histone 2B IgG autoreactivity in different population groups

Correlation between ELISA using native compared to PAD4-citrullinated full-length bovine histone 2B for detection of serum IgG autoantibodies. In the APCA negative RA patients (**B**) and the population controls (**C**) stronger correlations between cit-His2B and nat-His2B were detected than in the ACPA positive RA (**A**). This correlation is likely explained by binding to native epitopes present in both antigens. One individual in the population control group (marked in red) had high citrullinated histone reactivity, however this individual was also highly CCP2 positive (CCPlus, Euro Diagnostica, 1040 RU). Spearman analysis p-valuse and R-values are presented.

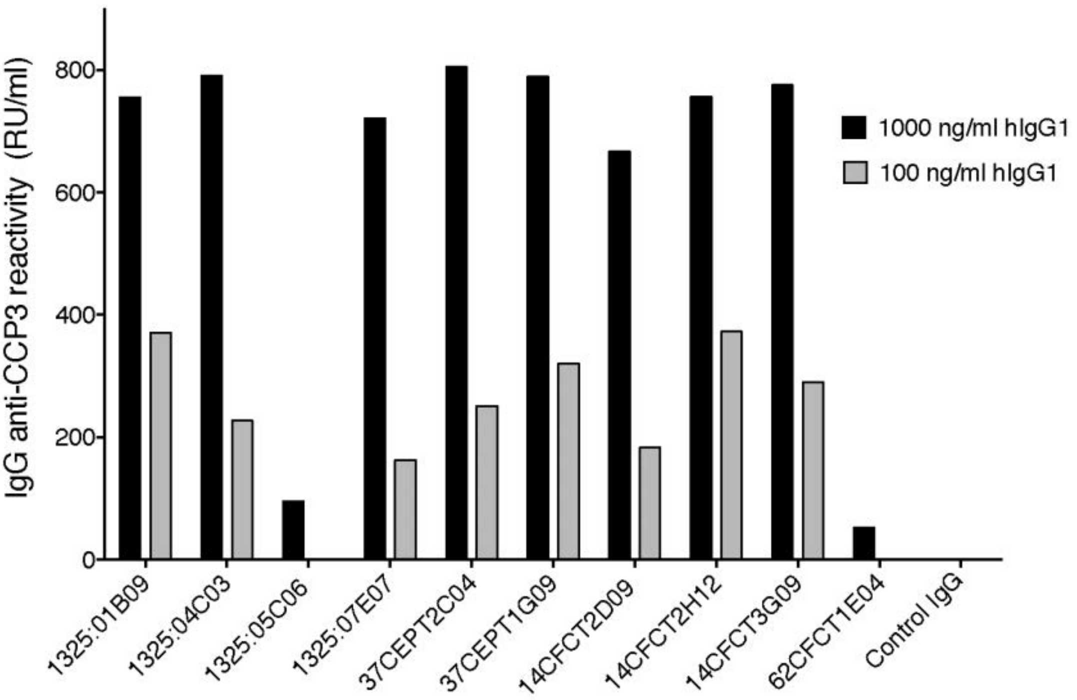

Supplementary Figure 2. CCP reactivity of monoclonal ACPA

The monoclonal recombinant ACPA hIgG1 were evaluated for binding to CCP with the commercial CCP3 kit (Inova Diagnostics) at 1000 ng/ml and 100 ng/ml. The RA-derived control IgG 1276:01G09 did not show any binding.

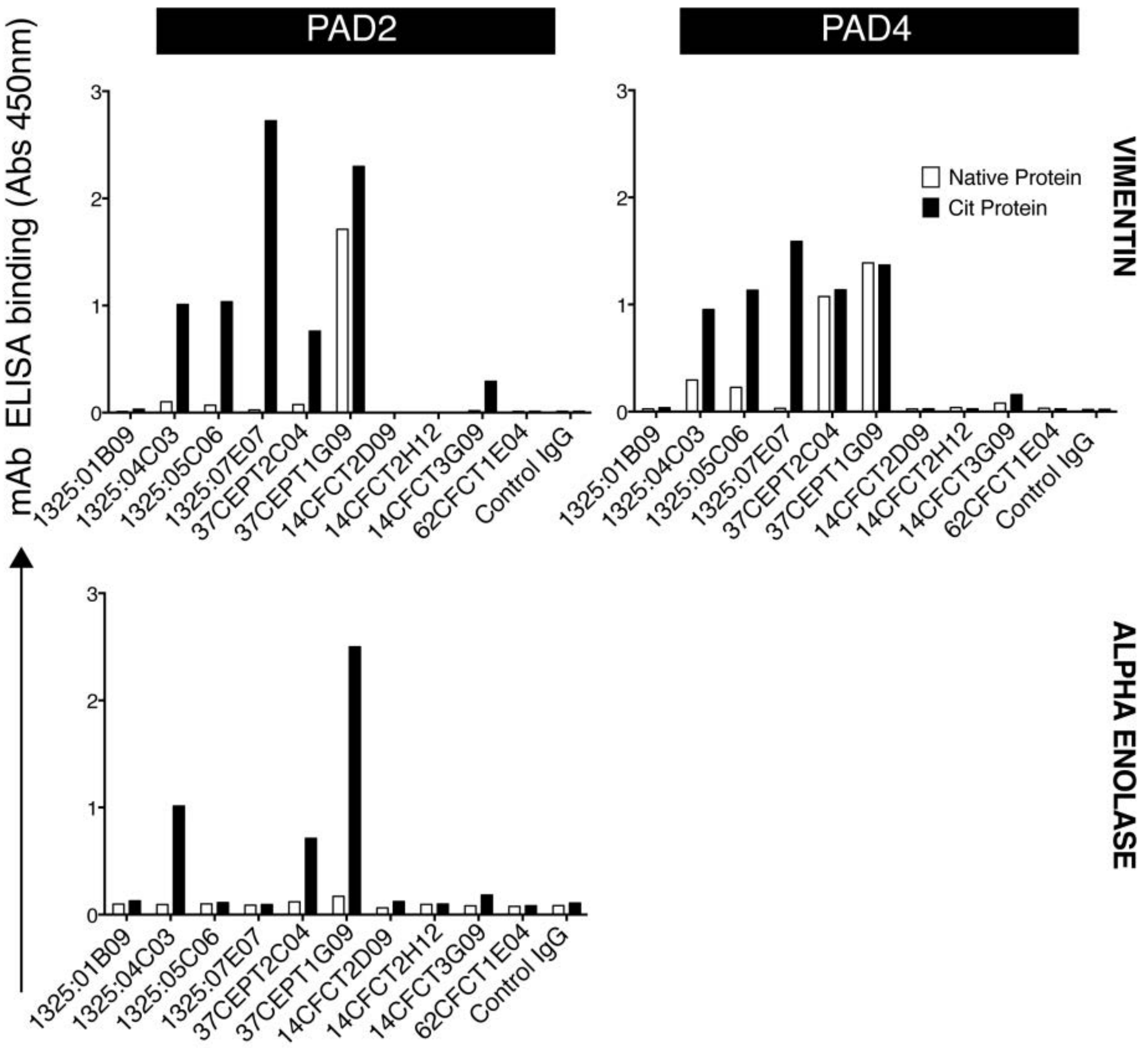

Supplementary Figure 3. ACPA mAb binding to citrullinated full-length cytoplasmic proteins.

Human monoclonal IgG1 ACPA were assessed for binding to full-length citrullinated human vimentin and alpha enolase by ELISA at 5 µg/ml. The proteins were coated to the ELISA plate and citrullinated by PAD2 or PAD4 under denaturing condition in solid-phase. The native proteins were control treated but without addition of the enzyme.

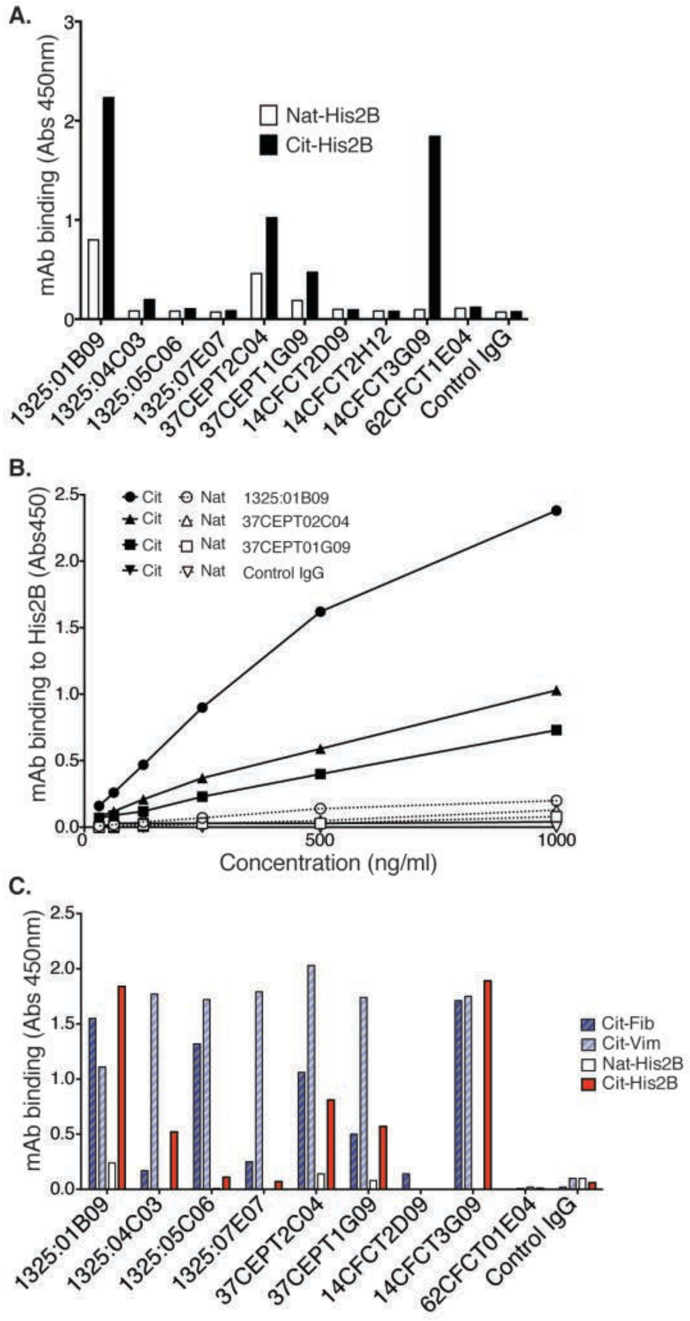

Supplementary Figure 4. ACPA mAb reactivity to in-solution citrullinated histone 2B.

(**A***)* Binding to citrullinated full-length Histone 2B was evaluated by ELISA. Bovine purified Histone 2B (Cit-His2B) was citrullinated by PAD4 in solution, buffer exchanged to PBS, and thereafter coated to ELISA wells. Native Histone (Native His2B) was control treated but without addition of the enzyme. ACPA IgG was assessed at 5 μg/ml and the figure depicts average values of duplicates. The RA derived non-ACPA mAb 1276:01G09 was used as control. (**B**) Serial dilutions of positive ACPA mAbs on citrullinated histone 2B and native histone 2B at indicated concentrations. (**C**) ACPA mAb ELISA reactivity to full-length citrullinated histone 2B compared to rabbit PAD citrullinated recombinant human vimentin and PAD4 citrullinated purified human fibrinogen.

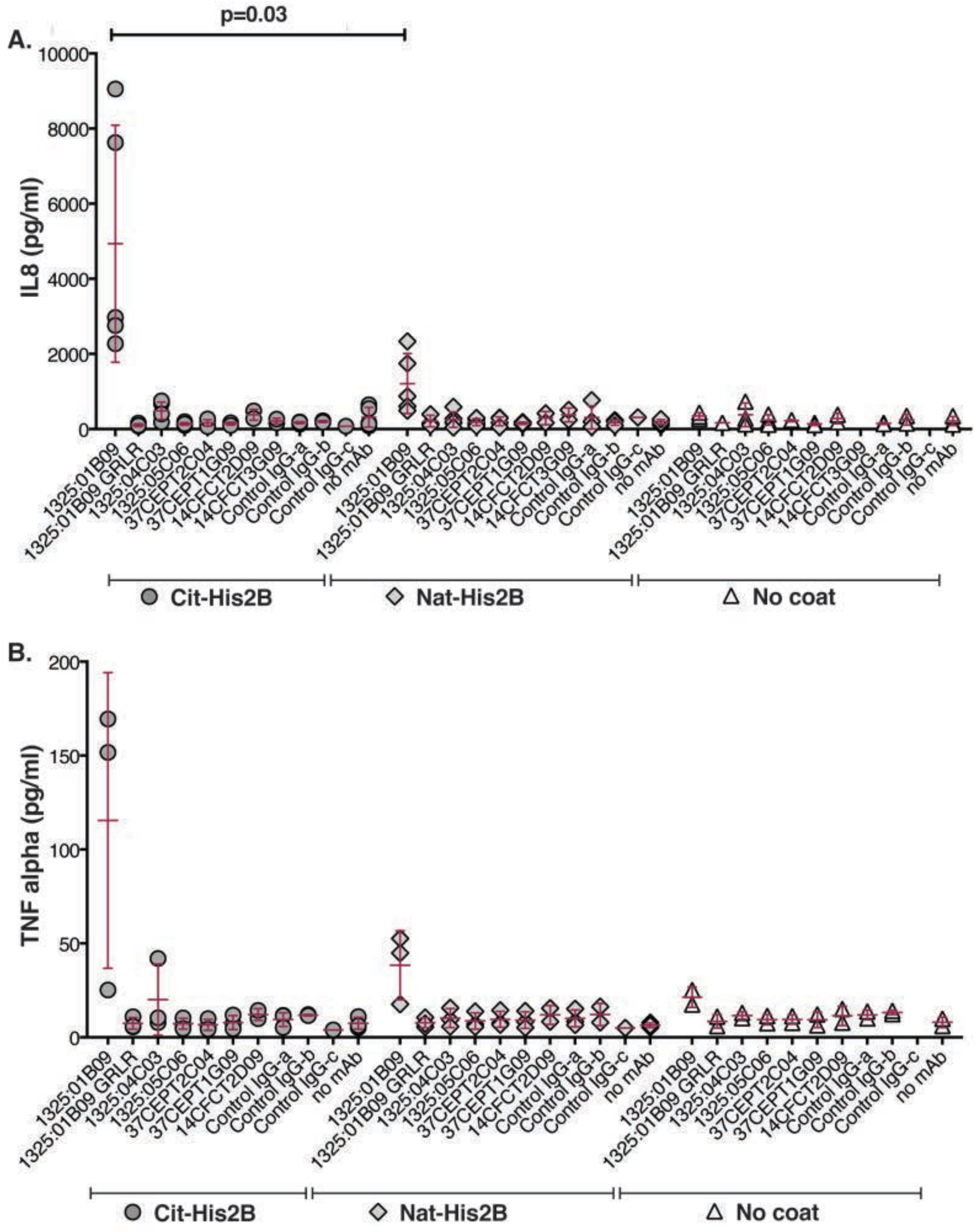

Supplementary Figure 5. The interaction between citrullinated histone and monoclonal ACPA induces cytokine responses

The immune stimulatory effect of monoclonal ACPA at 10 µg/ml was evaluated using plate-bound immune complexes with PAD4 citrullinated histone 2B. Healthy donor PBMC was incubated with the antigen-captured IgG plates for 20 hrs at 37°C and IL8 (**A**) and TNF alpha (**B**) expression was subsequently assessed in the cell supernatants by ELISA. Both recombinant RA-derived human IgG, 1362:01H05 or 1276:01G09 (Control IgG-a Control IgG-b, respectively), and commercial isotype control IgG (ET901, Biolegend, Control IgG-c) were used as controls. The figure shows average values of triplicate in different donors. P-values are derived from students t-test.

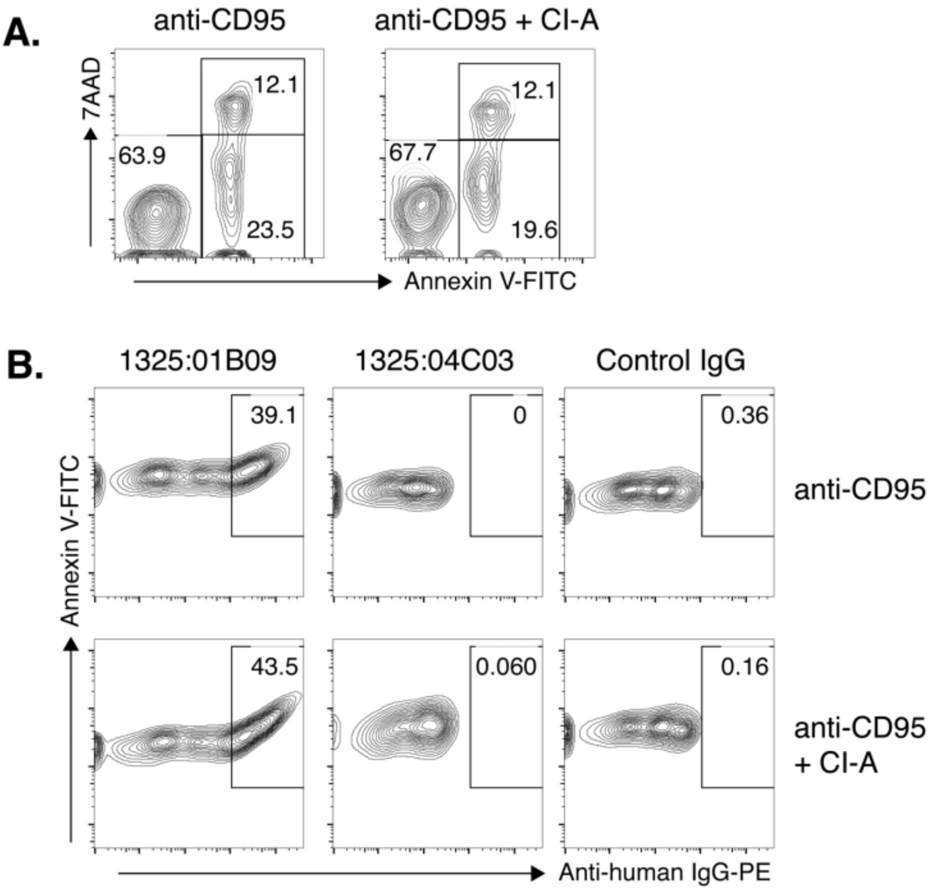

Supplementary Figure 6. Pharmacological PAD inhibition did not affect ACPA apoptotic cell binding

Flow cytometry analysis of ACPA hIgG1 mAb binding to Jurkat cells treated with the PAD inhibitor chlor-amidine (Cl-A, 20 μM) for 30 min, followed by induction of apoptosis with anti-CD95/Fas for 2 hrs (100 ng/ml) 37°C. (**A**) Cl-A treatment did not significantly change the level of apoptosis assessed by 7AAD/Annexin V staining. (**B**). Binding of mAbs to late apoptotic cells (7AAD+ Annexin V+ cells) were evaluated at 10 μg/ml.

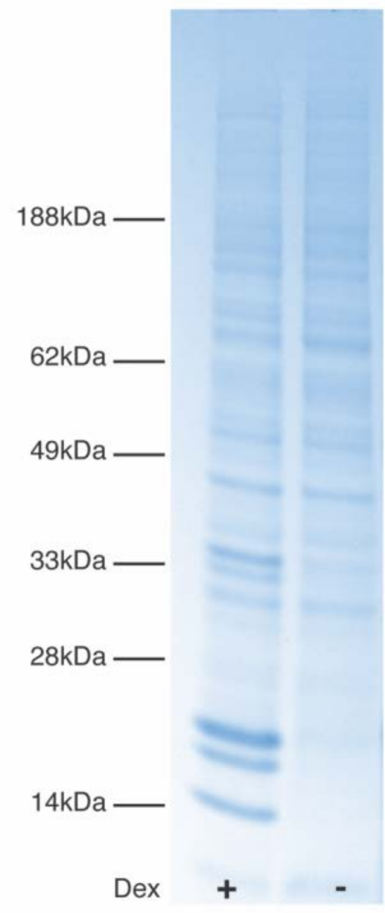

Supplementary Figure 7. Release of histone proteins in apoptotic cells.

The figure shows SDS-PAGE separated and Safe-Coomassie SimplyBlue SafeStain visualization of 10 µg cell lysates from apoptotic murine thymocytes treated with dexamethosone for 4 hrs (Dex +) or control treated cells (Dex -). An increased released of low molecular bands hypothesized to be histones can be seen in the apoptotic cells.

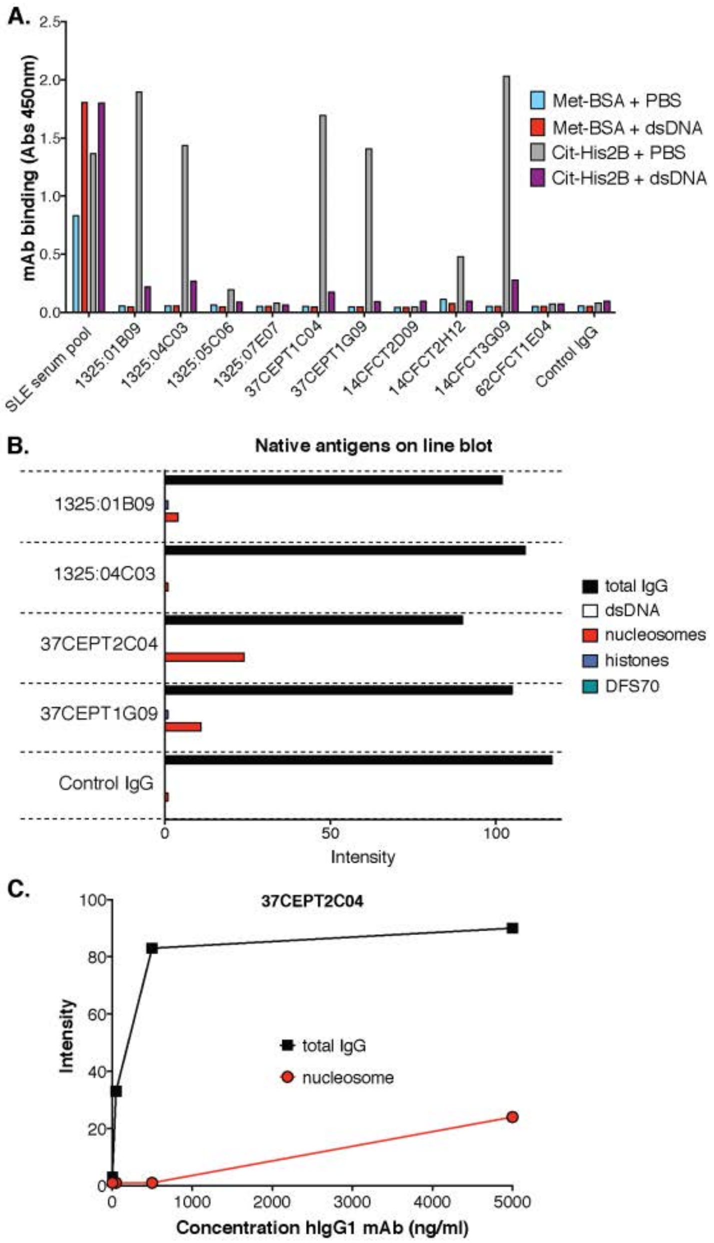

Supplementary Figure 8. ACPA mAbs did not significantly bind to dsDNA or nucleosomes.
The monoclonal ACPA (hIgG1 at 5 µg/ml) did not show any binding to native nuclear antigens using the methylated BSA capture dsDNA ELISA or commercial clinical line blot assay (EUROLINE ANA profile 3, Euroimmune). (**A**) Results from surfaces coated either with methylated BSA 5 µg/ml (met-BSA + PBS), methylated BSA 5 µg/ml and subsequent capture of calf thymus activated dsDNA 50 µg/ml (met-BSA + dsDNA), PAD4-citrullinated histone 2B 5 µg/ml (Cit-His2B + PBS), or cit-His2B and subsequent capture of dsDNA 50 µg/ml (Cit-His2B +dsDNA). Notably, dsDNA blocked binding of the ACPA to citrullinated histone epitopes and the ACPA showed no binding to dsDNA. (**B**) Plotted intensities from the line blot ANA assay (Euroimmune) including purified dsDNA, nucleosomes, and histones, as well as recombinant DSF70 protein. Anti-IgG is used as a positive control to detect total IgG. Only the clone 37CEPT2C04 showed weak anti-nucleosome reactivity at 5 µg/ml. (**C**) Titration of 37CEPT2C04 IgG towards nucleosomes in the line blot assay at indicated concentration with anti-IgG reactivity as control.

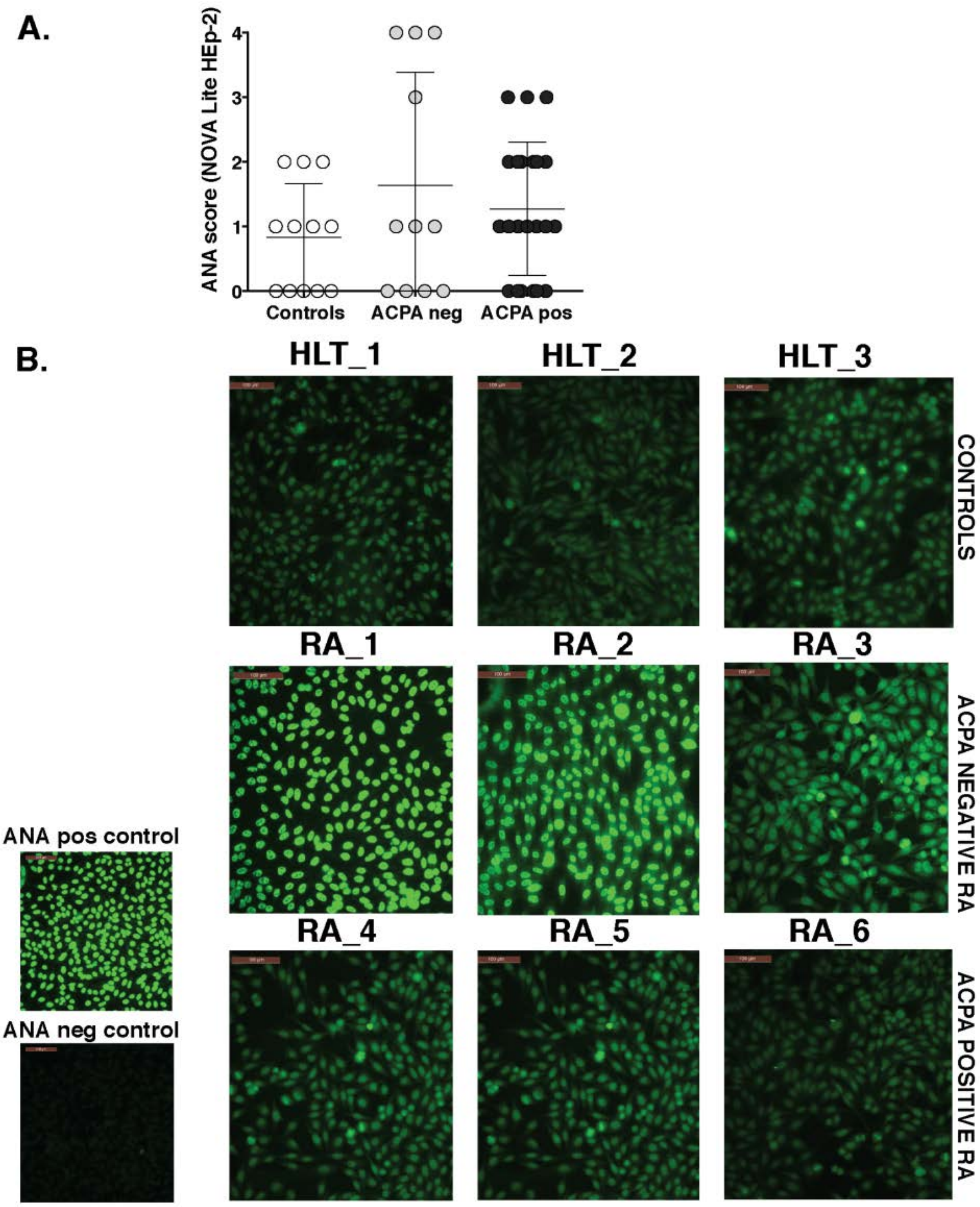

Supplementary Figure 9. ANA reactivity in RA patients

ANA reactivity was assessed in 12 population controls, 11 seronegative RA patients, 22 seropositive RA patients, using the Inova Lite ANA HEp-2 kit (Inova Diagnostics) and diluting the serum samples 1:40. (**A**) Summary of ANA scores at 1:40 dilution**.** (**B**) Examples of three individuals with positive staining from each of the groups (controls, ACPA positive RA, and ACPA negative RA).

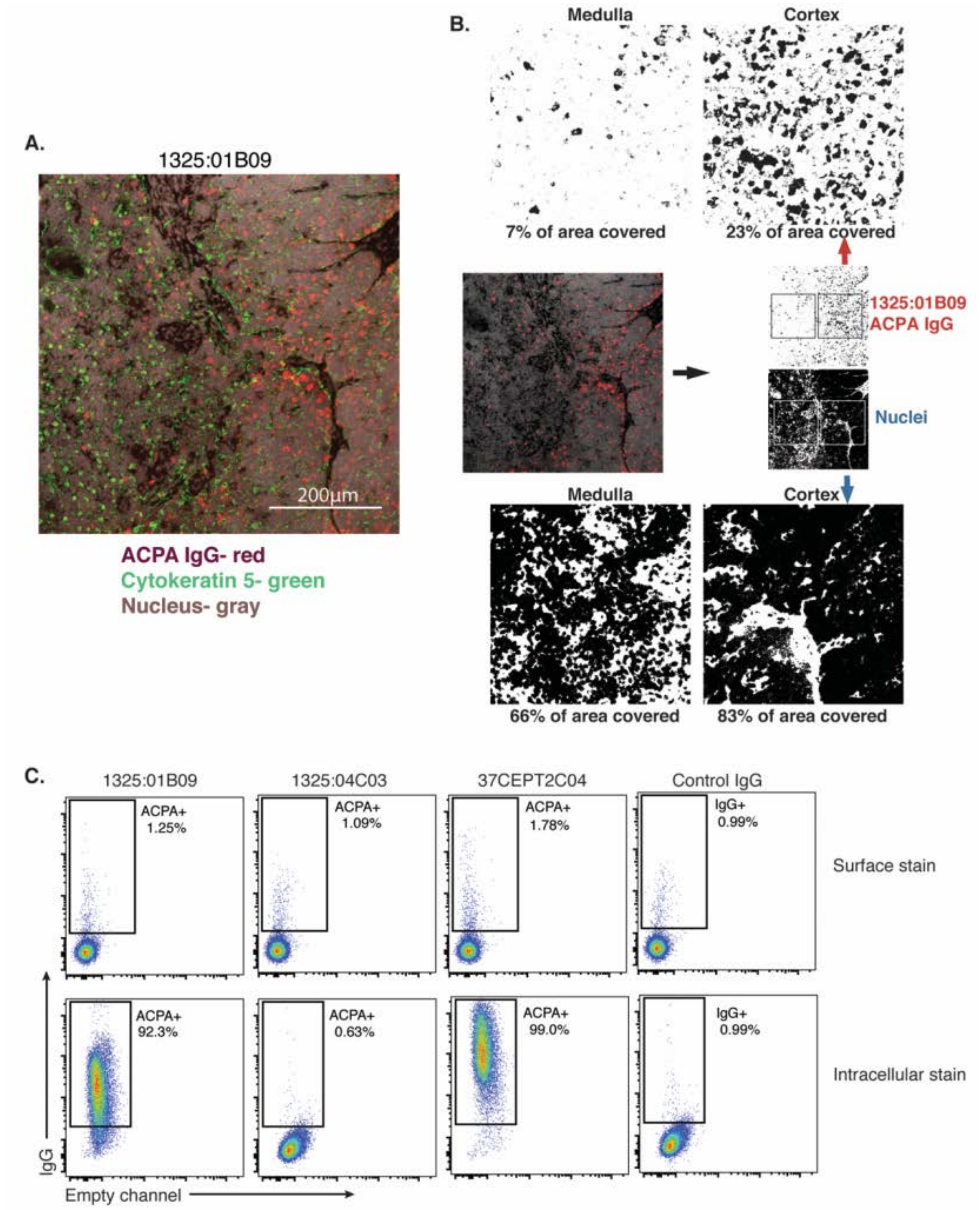

Supplementary Figure 10. ACPA intracellular binding to human primary thymocytes

(A) Immunofluorescent staining showing binding of the monoclonal ACPA 1325:01B09 (5 µg/ml biotinylated IgG) to human thymus tissue. IgG binding is visualized in red and medullary epithelial cells by cytokeratin 5 in green. The nuclear blue Hoechst staining has in this image been exchanged for gray to improve ACPA visualization. (**B**) Representative image quantification of 1325:01B09 ACPA IgG staining and nuclear staining in the medulla compared to cortex area. (**C**) ACPA IgG binding to human thymocytes was evaluated by flow cytometry. Single cell suspensions of human thymus tissue were treated with and without with Transcription Factor Staining Buffer Set (Life Technologies) to fix and permeabilize cells, and ACPA binding was assessed at 10 µg/ml biotinylated monoclonal IgG followed by detected with SAV-APC. Dead cells were excluded by fixable viability dye 506 (BD Bioscience).

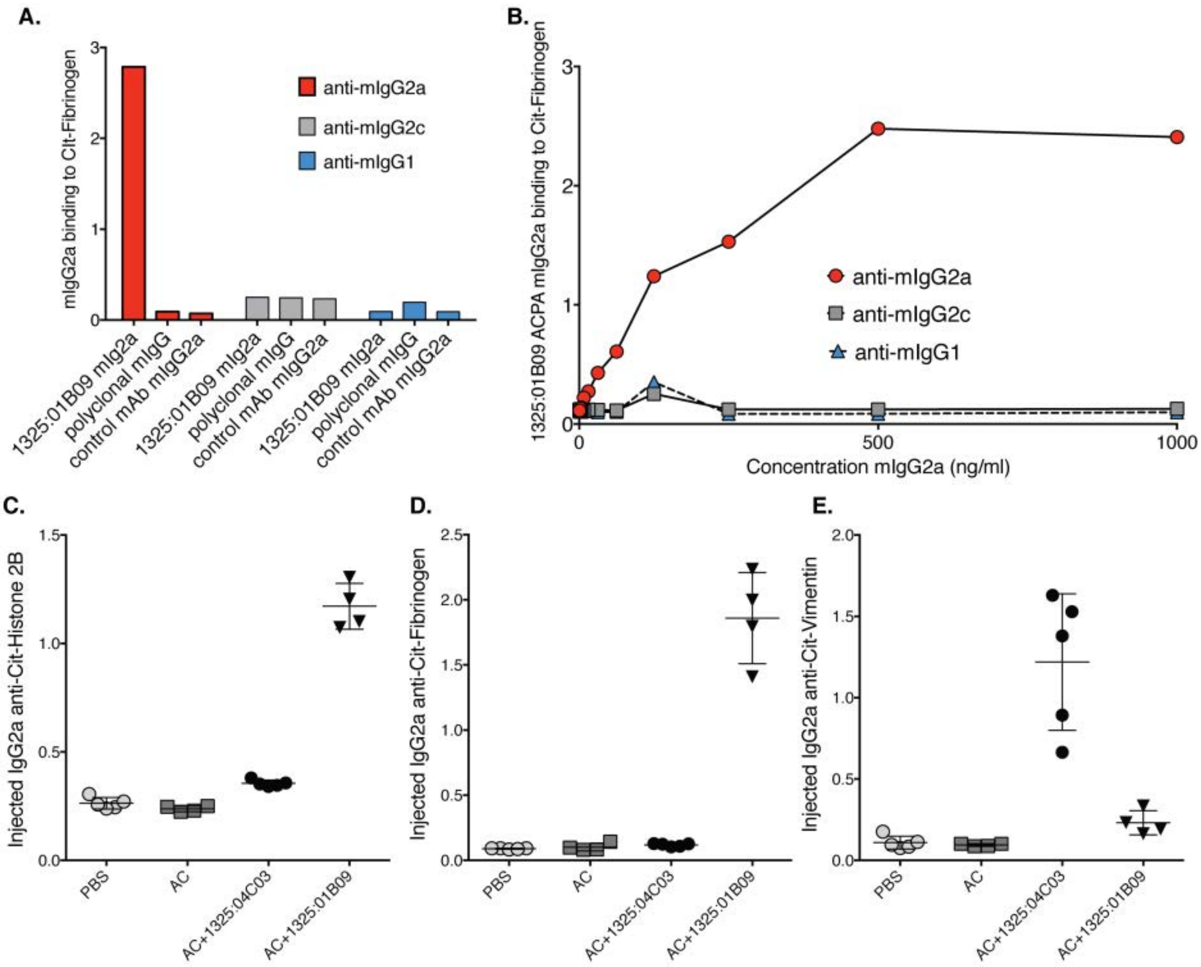

Supplementary Figure 11. Detection of injected ACPA IgG2a mAbs in C57BL/6 mice in a murine model of apoptotic cell mediated autoimmunity

(A-B) Evaluation of subclass specific HRP conjugated goat anti-mouse IgG2a, IgG2c, or IgG1 (Southern Biotech) detection reagents in ELISA. We utilized binding to citrullinated fibrinogen of the ACPA clone 1325:01B09 as mIgG2a at 5 µg/ml (**A**) or at different concentration (**B**). No significant cross-reactivity of the anti-IgG2c and anti-IgG1 reagents was observed. **(C-D)** Serum levels of injected ACPA IgG2a in C57BL/6 mice treated with apoptotic cells in combination with ACPA IgG measured by subclass specific ELISA. The injected ACPA were detected by PAD4 citrullinated full-length protein ELISA at day 5 after the first injection in serum at 1:100 dilution. Notably, the 1325:01B09 clone binds citrullinated histone (**C**) and fibrinogen (**D**) and not vimentin (**E**) while 1325:04C03 binds only to citrullinated vimentin and not citrullinated fibrinogen and histone. The figure shows absorbance at 450 nm.

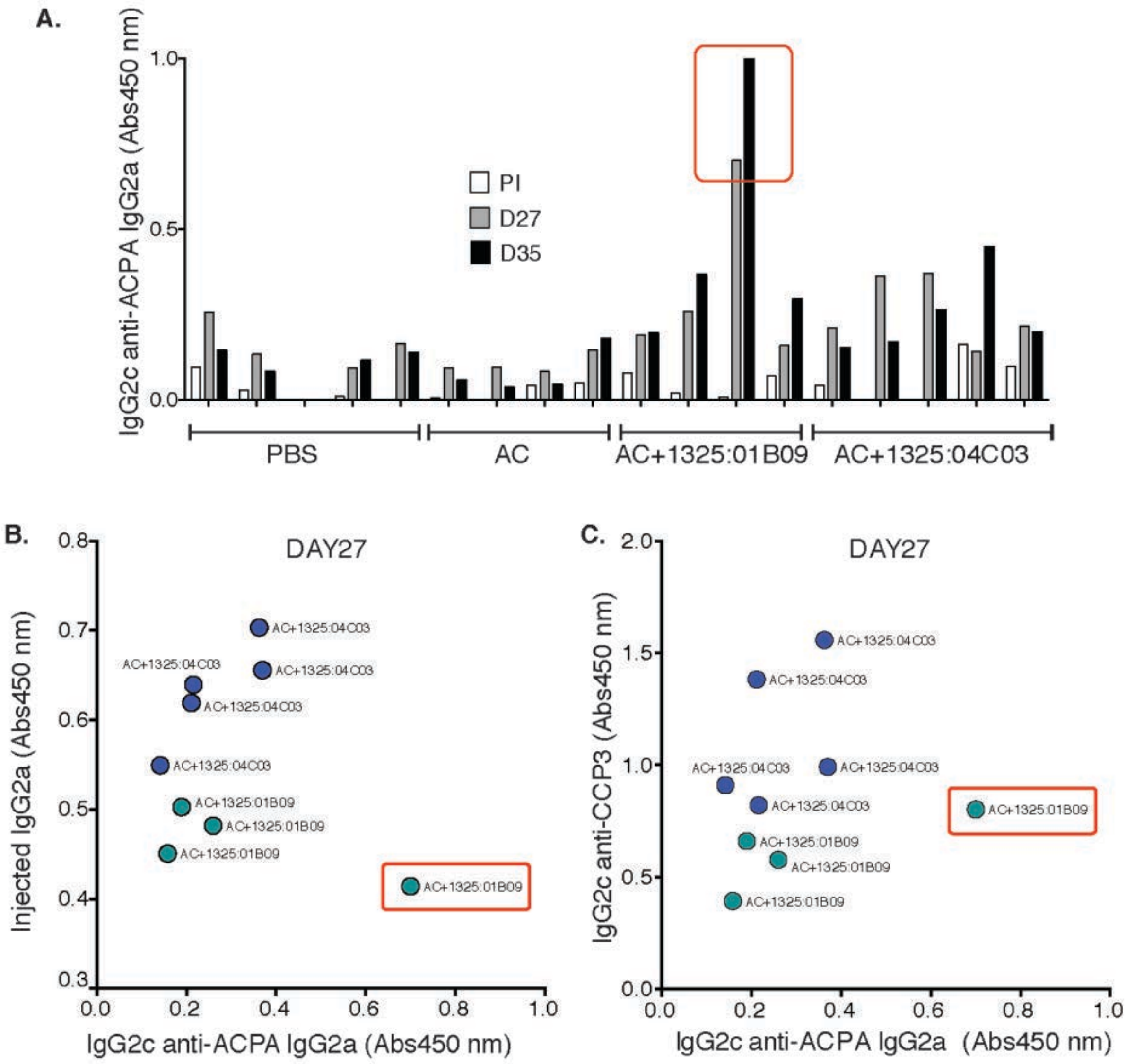

Supplementary Figure 12. Immunogenicity of murine IgG2a chimera in C57BL/6 mice (**A**) ELISA measurements of IgG2c to murine chimera IgG2a ACPA mAbs. ELISA wells were coated with equal amounts of 1325:01B09 and 1325:04C03 mIgG2a (3 + 3 µg/ml) and IgG2c reactivity was measure in serum diluted 1:100. Some immune response was detected in the mice that had been injected with mAbs and apoptotic cells (AC) and especially one mouse (marked in red) showed a significant response. No significant correlation was seen between anti-mIgG2a response and total concentration if circulating injected antibody (**B**) or with IgG2c anti-CCP3 reactivity (**C**).

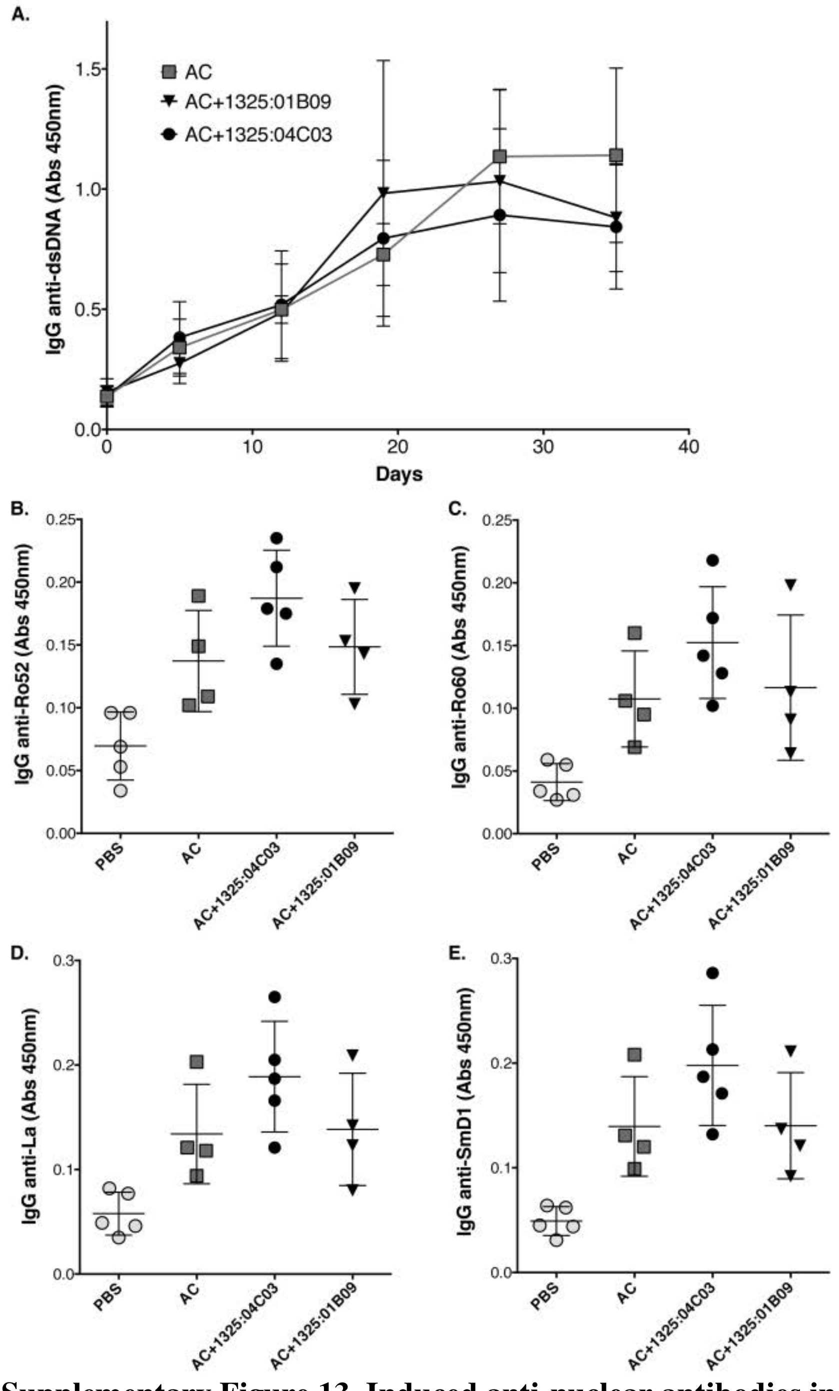

Supplementary Figure 13. Induced anti-nuclear antibodies in a murine model of apoptotic cell mediated autoimmunity

IgG reactivity to nuclear antigens were assessed with ELISA in serum at dilution 1:50 (anti-dsDNA) or 1:100 (anti-Ro52/60/La/SmD1). Mice were injected with 10^7 apoptotic cells at day 1, 7, 14, and 21. The first and 3rd injections were given in combination with 1 mg ACPA mIgG2a. (**A**) Anti-dsDNA response over time. (**B-E**) IgG anti-Ro52, anti-Ro60, anti-La, or anti-SmD1, at day 27.

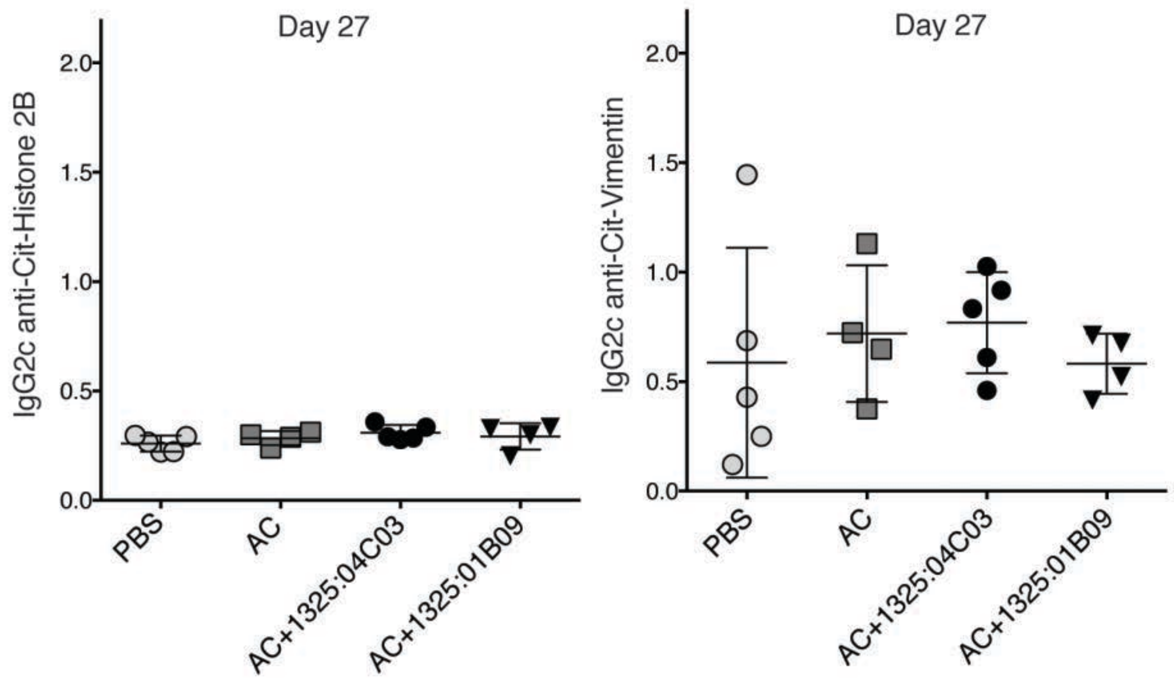

Supplementary Figure 14. Induced anti-citrullinated vimentin and histone in a murine model of apoptotic cell mediated autoimmunity

IgG2c reactivity to full-length rabbit PAD citrullinated human purified vimentin and PAD4 citrullinated purified bovine histone 2B were assessed with ELISA in serum at dilution 1:100 using subclass specific detection. Mice were injected with 10^7 apoptotic cells at day 1, 7, 14, and 21. The first and third injections were given in combination with 1 mg ACPA mIgG2a. No reactivity was observed at day 27.

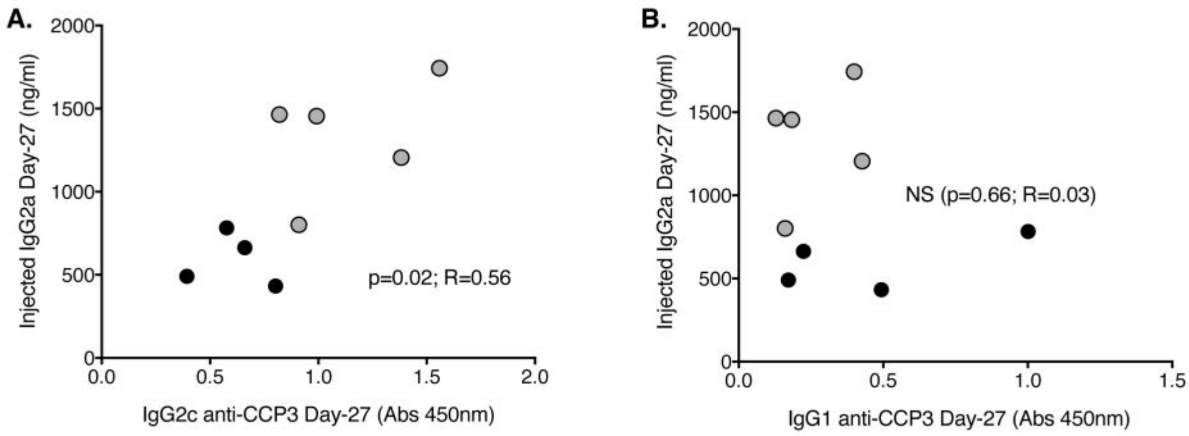

Supplementary Figure 15. Correlation between circulating levels of injected ACPA IgG2a mAbs and induced anti-CCP3 murine responses in a murine model of apoptotic cell mediated autoimmunity

Circulating injected ACPA IgG2a was correlated with IgG2c **(A)** or IgG1 **(B)** anti-CCP3 (modified assay from Inova Diagnostics) levels at day 27 after the first injection. 1325:01B09 injected mice are shown in black circles and 1325:04C03 injected mice are shown in gray. Mice were injected with 10^7 apoptotic cells at day 1, 7, 14, and 21. The first and 3rd injections were given in combination with 1 mg ACPA mIgG2a. P-values and R-values are shown from Pearson correlation.

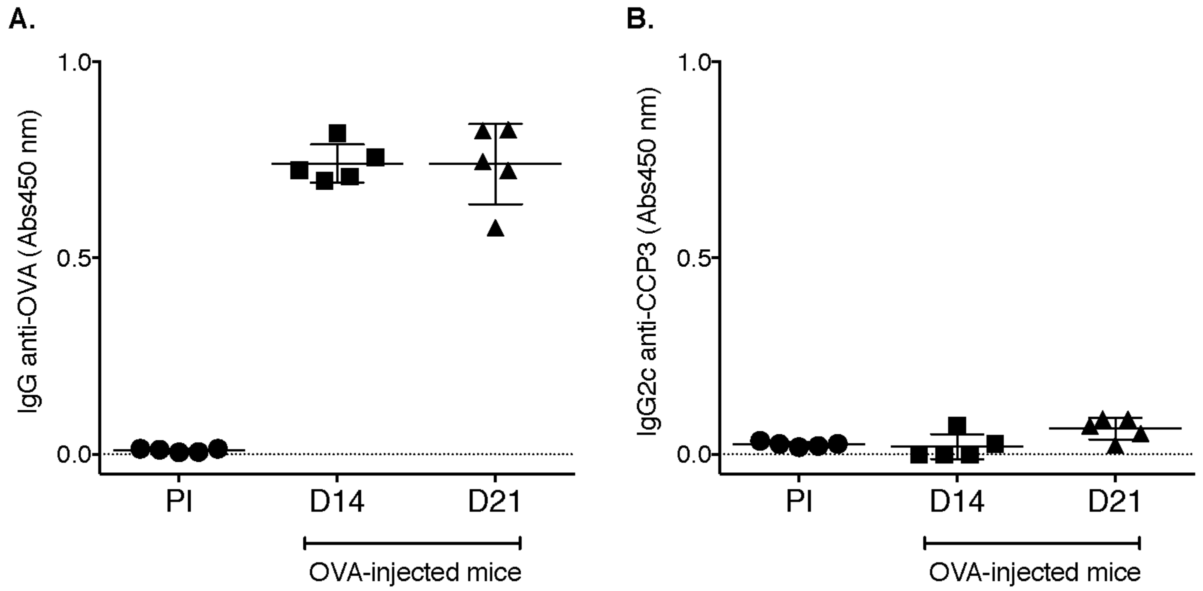

Supplementary Figure 16. No CCP3 reactivity were detected in OVA immunized mice

Serum IgG2c anti-CCP3 reactivity was measured by ELISA using the CCP3 coated plates (Inova Diagnostics) and subclass specific detection (Southern Biotech) in C57BL/6 mice immunized with ovalbumin/alum pre-injection (PI) and on day 14 (D14) and day 21 (D21) after immunization. IgG anti-OVA(**A**) and IgG2c anti-CCP3 (**B**) was measured in serum diluted 1:100.

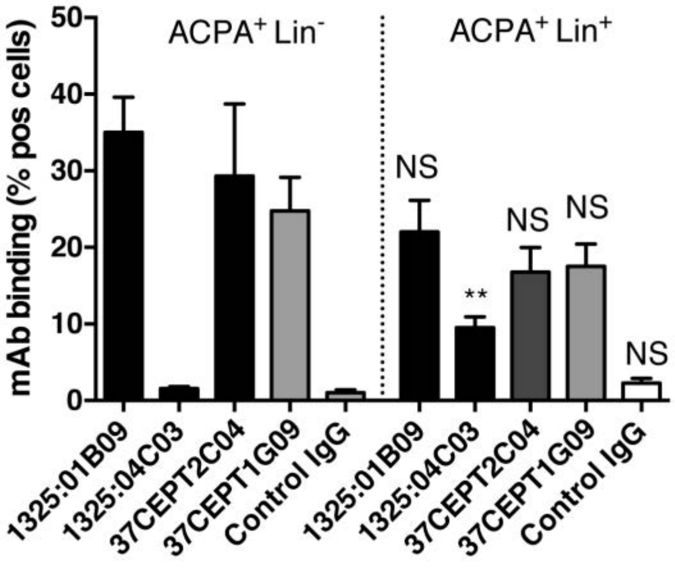

Supplementary Figure 17. ACPA binding to primary murine bone marrow cells

Flow cytometry binding to permeabilized murine precursor cells (Lin-) or differentiated bone marrow cells (Lin+). The linage detection panel (Milenyi Biotec) for differentiated cells included antibodies against CD5, CD11b, CD45R, Ly-6B.2 (7-4), Ly6G/C and Terr-119. The figure shows results (Mean and SD of % binding) for staining of bone marrow from four Balb/c mice, in one representative experiment. ** p-value 0.01-0.001 derived from student t-test comparing binding to Lin- and Lin+ cells for each mAb.

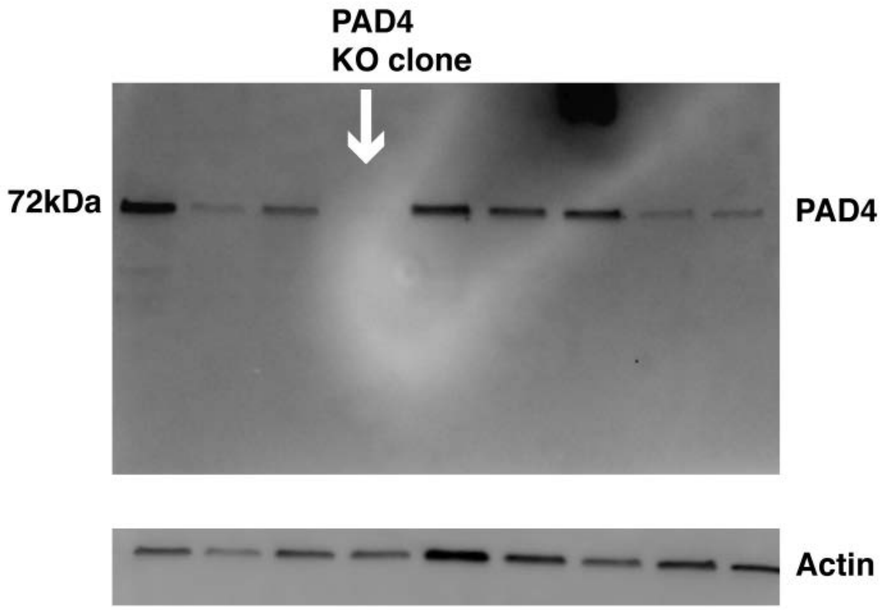

Supplementary Figure 18. CRISPR-KO of PAD4

PAD4 KO in ECoM-G murine neutrophils was confirmed with Western blot analysis using a specific PAD4 detection antibody (Abcam ab214810). The western blot shows successful CRISPR-CAS9 PAD4 KO in the fourth clone from the left, referred to in all further experiments as ECoM-G PAD4 KO.

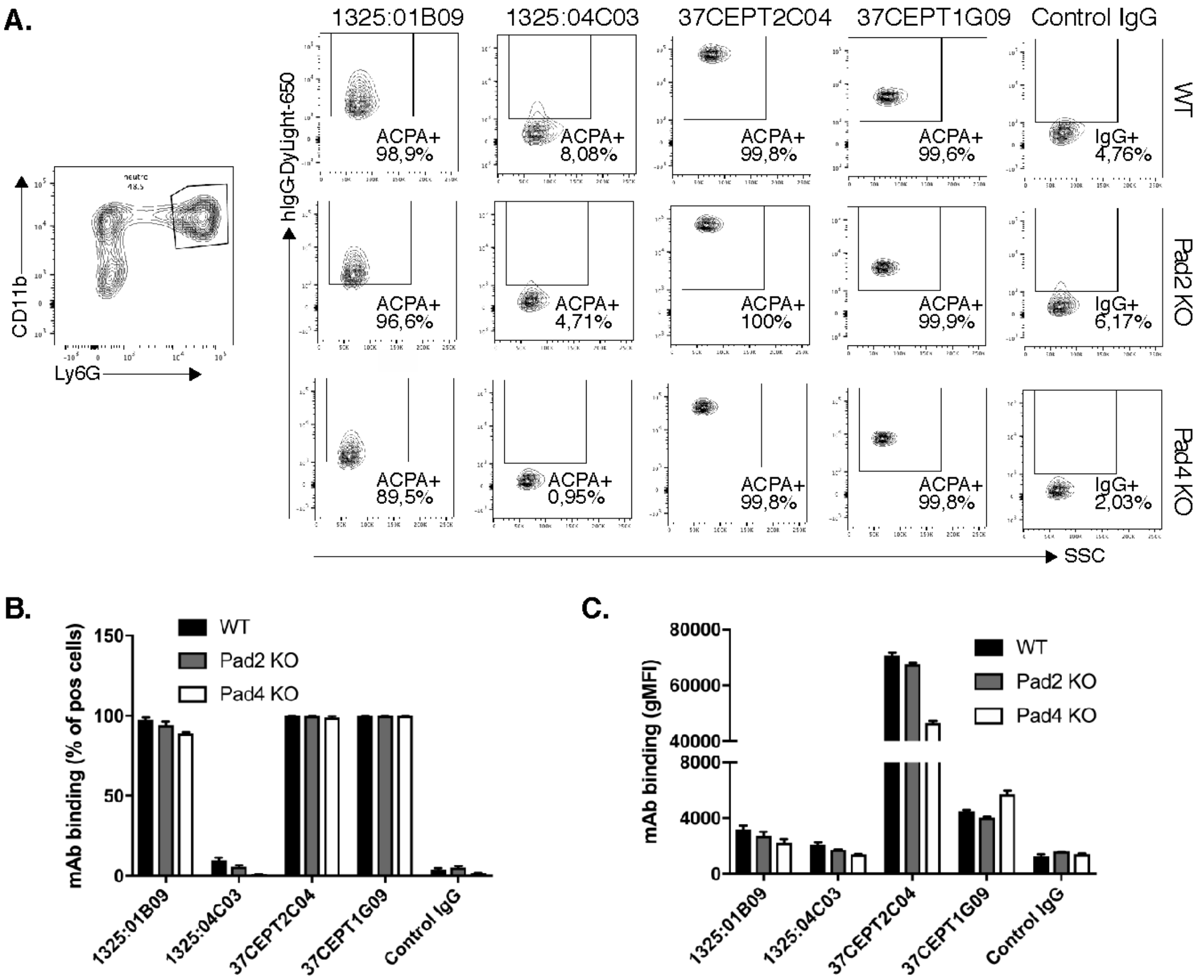

Supplementary Figure 19. Anti-nuclear ACPA binding to mouse neutrophils is not dependent on PAD2 or PAD4

Flow cytometry binding of human ACPA monoclonal antibodies (10 µg/ml DyLight-650 hIgG1) to ionomycin stimulated (1 µM 1 h) permeabilized bone marrow neutrophils (Ly6G+ CD11b+) from wild type (FVB), PAD2-/-, or PAD4-/- mice (**A**). Flow cytometry data showed and as % binding (**B**) as geometric mean fluorescence for the DyLight-650 hIgG1 gate (**C**). The figure shows representative images from two repeat experiments.

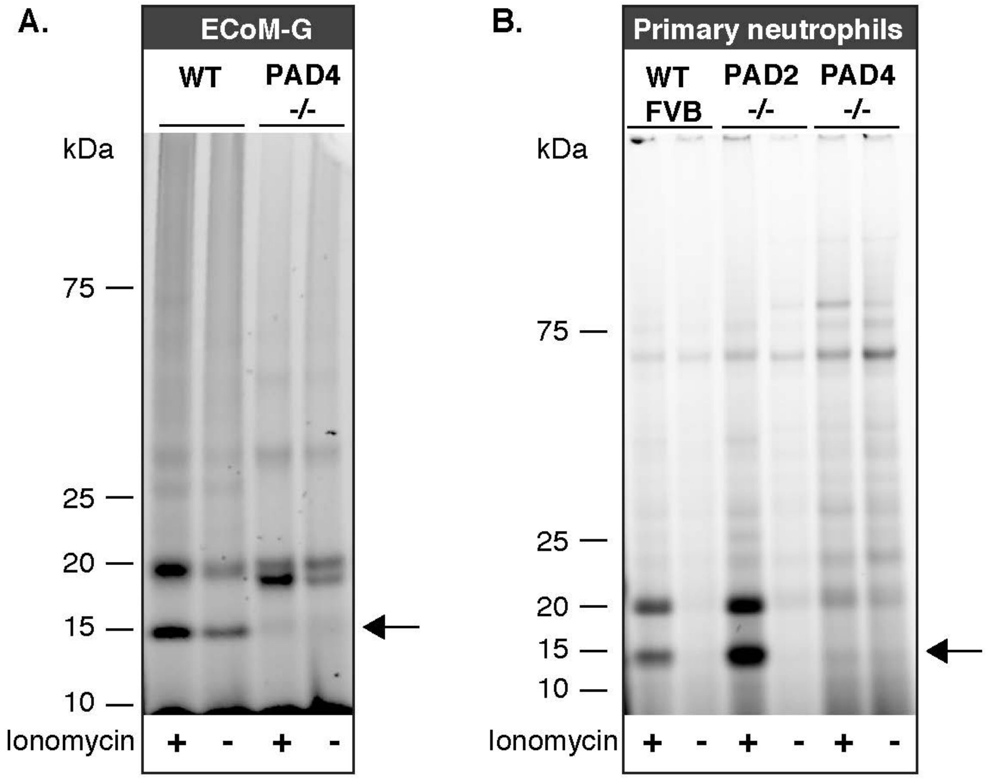

Supplementary Figure 20. Reduction in citrullination of PAD4 KO neutrophils
A reduction of citrullinated histones in PAD4 KO neutrophils could be confirmed by analysis of whole cell lysates using the rhodamine-based chemical citrulline-probe (Cayman Chemicals). (A) Lysates from CRISPR-CAS9 PAD4 KO ECoM-G murine neutrophils (B) Lysates from bone marrow neutrophils (Ly6G+ purified) from wild type (FVB), PAD2-/-, or PAD4-/- mice. Cells were stimulated with 1 µM ionomycin for 1 hr, before the cells were lysed with RIPA buffer and the lysates treated with acid and the citrulline-probe and separated on SDS-PAGE.

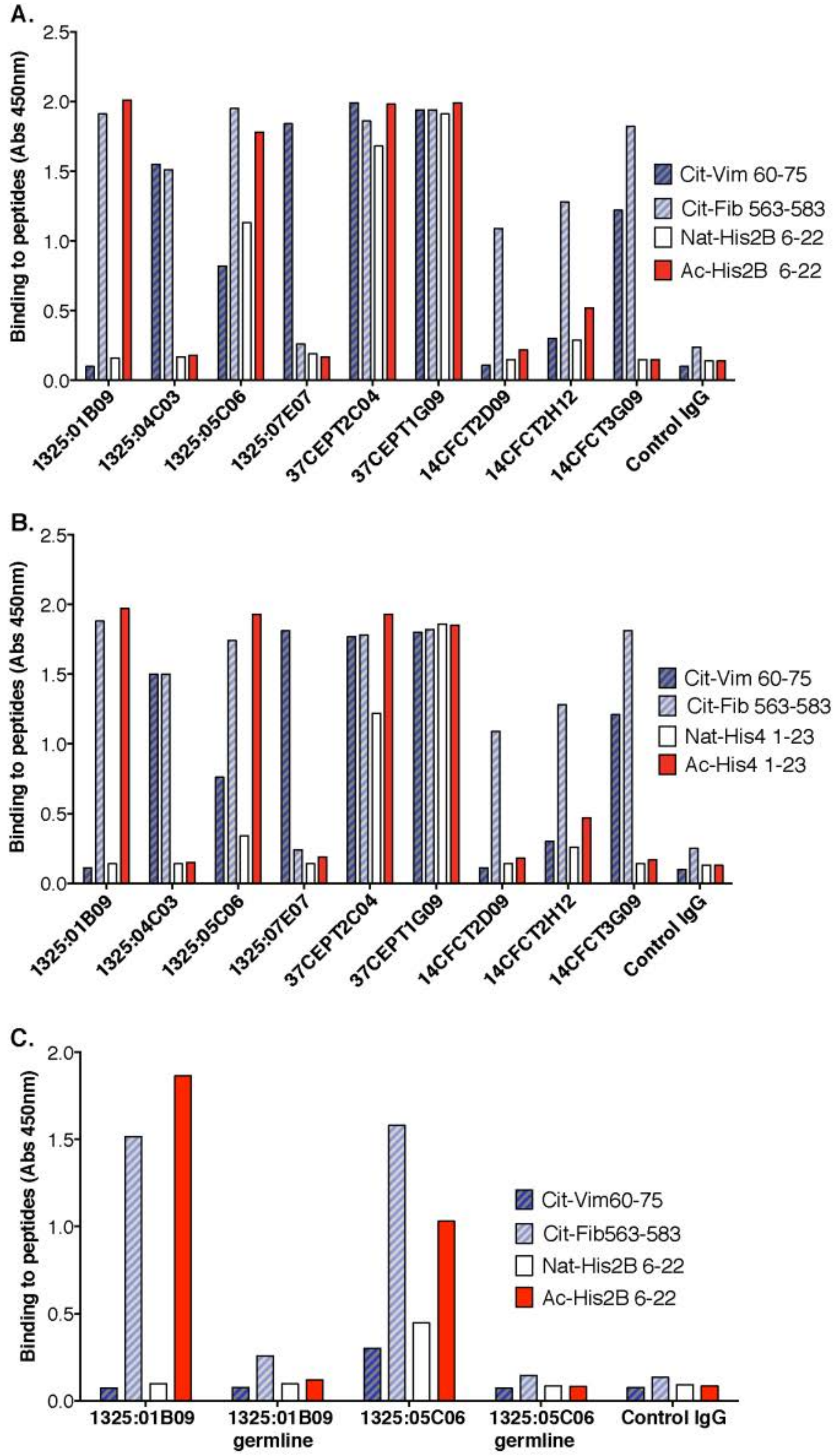

Supplementary Figure 21. ACPA mAb reactivity to acetylated histone peptides compare to citrullinated peptides

Reactivities of monoclonal ACPA mAbs to the acetylated histone 2B peptide (Ac-His2B 6-22) (**A**) or the acetylated histone 4 peptide (Ac-His4 1-23) (**B**) were compared to the respectively native lysine containing peptides and two citrullinated peptides from vimentin (cit-Vim60-75) and fibrinogen alpha chain (cit-Fib563-583) on the same ELISA plates. (**C**) Reactivity to Ac-His2B 6-22 of 1325:01B09 and 1325:05C06 mAbs converted to the closest germline encoded V(D)J rearrangements.

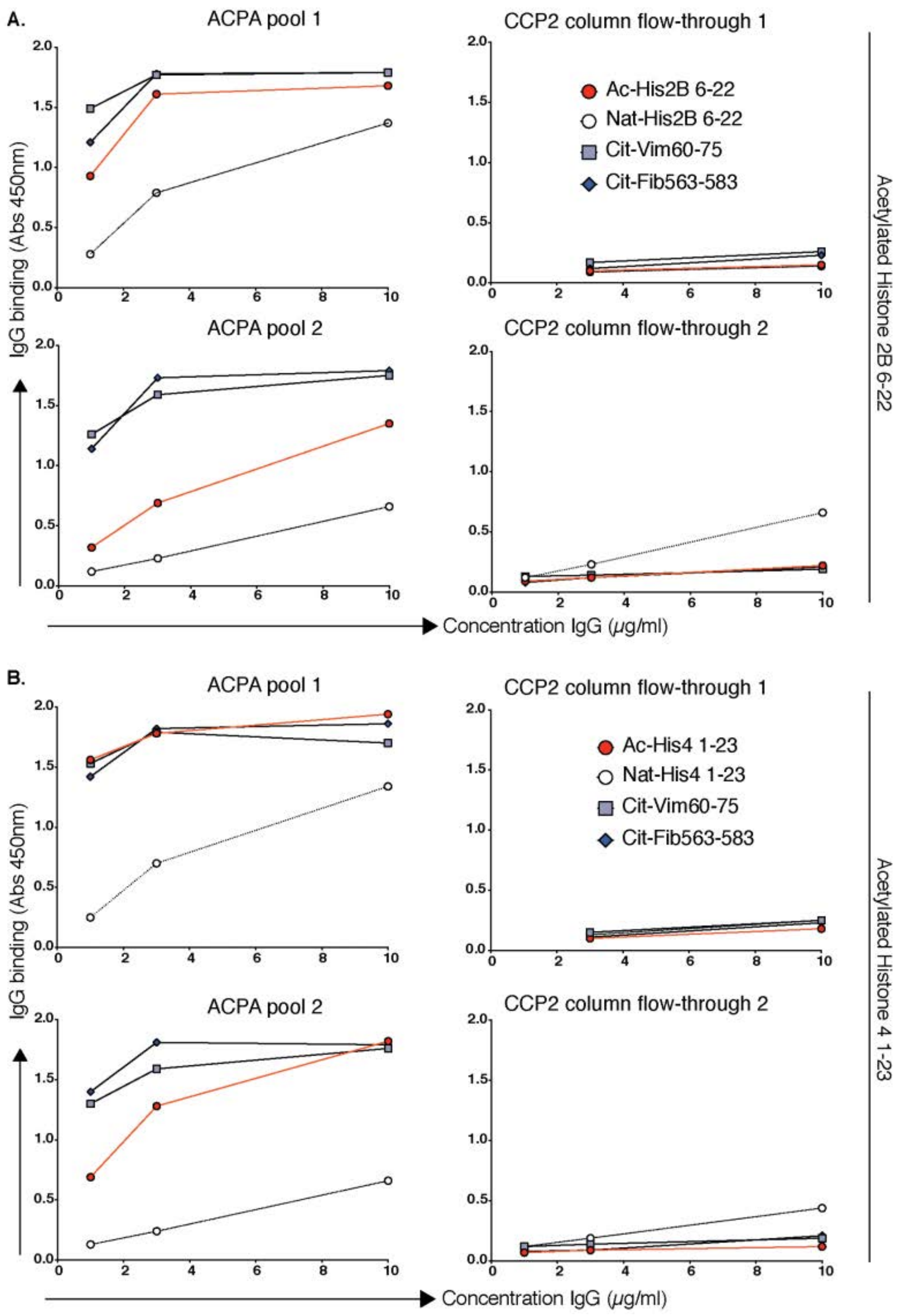

Supplementary Figure 22. Reactivity of human polyclonal ACPA to acetylated histone peptides

Two different polyclonal IgG ACPA pools were evaluated for reactivities to the acetylated histone 2B peptide (Ac-His2B 6-22) (**A**) or the acetylated histone 4 peptide (Ac-His4 1-23) (**B**) compared to the respectively native lysine containing peptides and two citrullinated peptides from vimentin (cit-Vim60-75) and fibrinogen alpha chain (cit-Fib563-583) on the same ELISA plates. The ACPA pools were purified by affinity chromatography with CCP2-columns (Euro Diagnostica) from serum IgG from 35 RA patients (ACPA pool 1) or from 108 RA patients (ACPA pool 2). The CCP2 column flow through is used as control.

Supplementary Table 1. Association of IgG autoreactivity to histone 2B in RA patients

	IgG anti-citrulline His 2B		IgG anti-native His 2B		Ratio cit/native	
	P-value#	R-value	P-value	R-value	P-value	R-value
Age	0.03	-0.14	0.002	-0.20	0.96	0.00
CRP	0.24	0.09	0.40	0.07	0.45	0.06
DAS28	0.33	0.08	0.63	-0.04	0.09	0.14
IgG anti-CCP2 ELISA	<0.0001	0.30	0.97	0.00	<0.0001	0.38
*IgG anti-**						
cit-Fil$_{307-324}$	<0.0001	0.38	0.20	0.08	<0.0001	0.42
arg-Fil$_{307-324}$	0.03	0.14	0.60	0.03	0.13	0.10
cit-Vim$_{60-75}$	0.0002	0.23	0.54	0.04	<0.0001	0.28
arg-Vim$_{60-75}$	0.78	-0.02	0.90	0.01	0.20	-0.08
cit-Vim$_{2-17}$	0.00001	0.27	0.33	0.06	<0.0001	0.29
arg-Vim$_{2-17}$	0.89	-0.01	0.91	-0.01	0.84	0.01
cit-Fibβ$_{36-52}$	<0.0001	0.44	0.05	0.13	<0.0001	0.45
arg-Fibβ$_{36-52}$	0.63	-0.03	0.15	-0.09	0.55	0.04
cit-Fibα$_{563-583}$	<0.0001	0.39	0.27	0.07	<0.0001	0.43
arg-Fibα$_{563-583}$	0.93	-0.01	0.86	0.01	0.75	-0.02
cit-Fibα$_{580-600}$	0.00006	0.25	0.84	-0.01	<0.0001	0.35
arg-Fibα$_{580-600}$	0.85	0.01	0.20	-0.08	0.11	0.10
cit-Fibβ$_{62-81a}$	0.11	0.10	0.77	-0.02	0.02	0.15
cit-Fibβ$_{62-81ab}$	0.004	0.19	0.25	0.07	0.01	0.17
arg-Fibβ$_{62-81a}$	0.56	-0.04	0.38	-0.06	0.53	0.04
cit-Eno$_{5-21}$ (CEP-1)	<0.0001	0.31	0.18	0.09	<0.0001	0.37
arg-Eno$_{5-21}$ (REP-1)	0.21	-0.08	0.16	-0.09	0.92	0.01

Results from Spearman correlation analysis of 243 RA patients (50 seronegative and 193 seropositive)

* Data generated by ISAC antigen micro array (34)

Supplementary Table 2. Mass-spectrometry identified proteins in ACPA mAb co-precipitation from apoptotic cell lysate

Accession	Description	Coverage [%]	Peptides [no.]	PSMs [no.]	Unique Peptides [no.]	MW [kDa]	Score Sequest HT
<12 kDa							
P62805	Histone H4	70	15	59	15	11.4	171.55
17-18 kDa							
P0C0S5	Histone H2A.Z	31	5	16	3	13.5	39.65
Q8N257	Histone H2B type 3-B	44	8	22	3	13.9	60.99
Q8IUE6	Histone H2A type 2-B	23	4	10	1	14	22.41
P0C0S8	Histone H2A type 1	41	8	39	2	14.1	120.12
Q93077	Histone H2A type 1-C	41	7	32	1	14.1	96.44
15-19 kDa							
P68431	Histone H3.1	37	7	21	7	15.4	50.46
B4DR52	Histone H2B	39	9	22	4	18	62.43
P84103	Serine/arginine-rich splicing factor 3	23	4	5	3	19.3	12.15
20-25 kDa							
P16403	Histone H1.2	26	9	20	1	21.4	65.67
P16402	Histone H1.3	22	9	20	1	22.3	65.59
Q92522	Histone H1x	17	3	3	3	22.5	7.47
P16401	Histone H1.5	22	9	22	8	22.6	76.76
Q9Y294	Histone chaperone ASF1A	26	3	4	3	23	13.21
> 30 kDa							
P06748	Nucleophosmin	17	4	5	4	32.6	13.52
O75367	Core histone macro-H2A.1	38	11	14	11	39.6	46.31
Q9H6F5	Coiled-coil domain-containing protein 86	16	4	4	4	40.2	13.09
P60709	Actin, cytoplasmic 1	39	10	12	4	41.7	35.73
Q09028	Histone-binding protein RBBP4	25	5	5	5	47.6	19.94
C9JC84	Fibrinogen gamma chain	20	4	5	4	52.3	19.91
F5H012	E3 ubiquitin-protein ligase TRIM21	25	10	13	10	54	41.9
P02675	Fibrinogen beta chain	10	3	3	3	55.9	13.04
P38646	Stress-70 protein, mitochondrial	35	18	27	18	73.6	82.94
P13010	X-ray repair cross-complementing protein 5	40	21	32	21	82.7	101.92

The ACPA monoclonal 1325:01B09 was used for co-precipitation from apoptotic Jurkat cells (treated with 25 μM etoposide overnight) and bound fraction was eluted with ammonium hydroxide.

The search was performed against Uniprot/Swissprot human sequence database (release 18.10.10) by ProteomeDiscoverer 2.2.0.388 using SEQUEST algorithm. The following parameters were used for the database search: tryptic digestion (maximum of 3 miscleavages); carbamidomethylation (C) as a fixed modification; oxidation (M), acetylation (K), deamination (N/Q) and citrullination (R) as the variable modifications; 10 ppm as the precursor tolerance; and 0.02 Da as the fragment tolerance.

PSM: The number of peptide spectrum matches. The number of PSM's is the total number of identified peptide spectra matched for the protein. The PSM value may be higher than the number of peptides identified for high-scoring proteins because peptides may be identified repeatedly.

Supplementary Table 3. Identified acetylated peptides in ACPA mAb co-precipitated from apoptotic cell lysate

Sequence	Modifications	Protein Groups	Proteins [no.]	PSMs [no.]	Protein Accessions	Protein Descriptions
AVT**K**VQK	1xAcetyl	1	3	1	B4DR52	Histone H2B
QLAT**K**AAR	1xAcetyl	1	9	4	P68431	Histone H3.1
STGG**K**APR	1xAcetyl	1	10	1	P68431	Histone H3.1
KQLAT**K**AAR	1xAcetyl	1	9	2	P68431	Histone H3.1
**K**QLAT**K**AAR	2xAcetyl	1	9	1	P68431	Histone H3.1
**K**SAPATGGVK	1xAcetyl	1	3	2	P68431	Histone H3.1
GLG**K**GGA**K**R	2xAcetyl	1	1	1	P62805	Histone H4
GG**K**GLG**K**GGAK	2xAcetyl	1	1	1	P62805	Histone H4
GG**K**GLG**K**GGA**K**R	3xAcetyl	1	1	2	P62805	Histone H4
G**K**GGKGLGK	1xAcetyl	1	1	2	P62805	Histone H4
G**K**GG**K**GLGK	2xAcetyl	1	1	1	P62805	Histone H4
G**K**GG**K**GLG**K**GGAK	3xAcetyl	1	1	1	P62805	Histone H4

The ACPA monoclonal 1325:01B09 was used for co-precipitation from apoptotic Jurkat cells (treated with 25 μM etoposide overnight) and bound fraction was eluted with ammonium hydroxide. No citrullinated peptides were identified.

The modified lysine (K) residues are shown in bold.

Identified acetylated lysines in the histone proteins:

Histone3.1 (P68431)
ARTKQTARKSTGG**K**$_{14}$APR**K**$_{18}$QLAT**K**$_{23}$AAR**K**$_{27}$SAPATGGVKKPHRYRPGTVALREIRRYQKS
TELLIRKLPFQRLVREIAQDFKTDLRFQSSAVMALQEACEAYLVGLFEDTNLCAIHAKRVTIM
PKDIQLARRIRGERA

Histone 2B (B4DR52)
PDPAKSAPAPKKGSKKAVT**K**$_{20}$VQKKDGKKRKRSRKESYSVYVYKVLKQVHPDTGISSKAMG
IMNSFVNDIFERIAGEASRLAHYNKRSTITSREIQTAVRLLLPGELAKHAVSEGTKAVTKYTSS
NPRNLSPTKPGGSEDRQPPPSQLSAIPPFCLVLRAGIAGQV

Histone 4 (P62805)
SGRG**K**$_{5}$GG**K**$_{8}$GLG**K**$_{12}$GGA**K**$_{16}$RHRKVLRDNIQGITKPAIRRLARRGGVKRISGLIYEETRGVLK
VFLENVIRDAVTYTEHAKRKTVTAMDVVYALKRQGRTLYGFGG
